# Pan-cancer analysis reveals differential PERK expression across tumour types and its potential as a therapeutic target

**DOI:** 10.1007/s12672-026-04910-8

**Published:** 2026-03-25

**Authors:** Shivam Kumar, Namshik Han, Georgia Tsagkogeorga, Murphy Lam Yim Wan

**Affiliations:** 1 School of Medicine, Pharmacy and Biomedical Sciences, Faculty of Science and Health, University of Portsmouth, Portsmouth, PO1 2DT UK; 2 Cambridge Centre for AI in Medicine, University of Cambridge, Cambridge, CB2 0QQ UK; 3 Milner Therapeutics Institute, University of Cambridge, Cambridge, CB4 0WS UK; 4 STORM Therapeutics Ltd, Babraham Research Campus, Cambridge, CB22 3AT UK; 5 Department of Laboratory Medicine, Division of Microbiology, Immunology and Glycobiology, Lund University, Lund, 221 84 Sweden

**Keywords:** Cancer, *EIF2AK3*, ER stress, PERK, Therapeutic target, Unfolded protein response

## Abstract

**Background:**

PERK (Protein kinase R-like endoplasmic reticulum kinase), encoded by *EIF2AK3*, is a key ER stress sensor that regulates protein synthesis and the unfolded protein response (UPR). Its dysregulation is linked to cancer development and progression. This study investigated *EIF2AK3*/PERK expression across 28 tumour types (*n* = 7,251) and their matched normal adjacent tissues (NATs; *n* = 667) from the Cancer Genome Atlas (TCGA) database, along with additional normal tissue samples from the Genotype-Tissue Expression (GTEx) project (*n* = 1,736), and 1,179 tumour cell lines, examining correlations with patient demographics, tumour stage, survival and genetic alterations.

**Results:**

Transcriptomic analysis revealed *EIF2AK3* upregulation in multiple cancers, with proteomic data supporting increased PERK protein levels in specific tumour types. *EIF2AK3* expression was correlated with age, gender, tumour stage and survival endpoints in a context-dependent manner. Mutational analysis identified frequent *EIF2AK3* alterations, including missense, gain and amplification events. Kyoto Encyclopedia of Genes and Genomes (KEGG) pathway enrichment analysis revealed that the cell cycle, proteoglycans in cancer and focal adhesion pathways were among the most consistently enriched across tumour types, particularly in tumours with high *EIF2AK3* expression. These tumours also showed enrichment in Rap1 signalling, PI3K-Akt signalling, cytoskeletal regulation, ubiquitin-mediated proteolysis and nucleocytoplasmic transport, indicating a stress-adaptive invasive phenotype. In contrast, tumours with low *EIF2AK3* expression demonstrated enrichment in focal adhesion, AGE-RAGE signalling and cytoskeletal organisation, suggesting a tumour state dependent on adhesion integrity and extracellular matrix (ECM) interaction rather than stress-induced plasticity. Gene Ontology (GO) enrichment analysis supported these observations, with high *EIF2AK3* tumours displaying strong associations with cell adhesion, ECM structural remodelling and actin binding, while low *EIF2AK3* tumours showed enrichment in cytoskeletal stability and axonogenesis. Hierarchical clustering revealed a distinct gene cluster associated with *EIF2AK3* across several tumour types. Protein-protein interaction (PPI) and enrichment analyses linked this cluster to tumour progression and cell adhesion.

**Conclusion:**

These findings underscore the central role of *EIF2AK3*/PERK in regulating cancer-associated pathways. Its expression stratifies tumours into biologically distinct phenotypes, with one favouring ER stress adaptation and invasiveness, and the other reliant on ECM organisation and adhesion homeostasis. *EIF2AK3* may thus serve as a potential therapeutic target in multiple cancers.

**Supplementary Information:**

The online version contains supplementary material available at 10.1007/s12672-026-04910-8.

## Background

The endoplasmic reticulum (ER) plays a pivotal role in maintaining cellular homeostasis and survival, including protein folding, lipid biosynthesis and calcium and redox homeostasis [1, 2]. When cells are exposed to unfavourable conditions such as hypoxia, nutrient deprivation, oxidative stress, or other metabolic disruptions, the delicate balance in the ER is disturbed, leading to the accumulation of misfolded or unfolded proteins within the ER lumen [3, 4]. This imbalance triggers ER stress and subsequently activates a cellular mechanism known as the unfolded protein response (UPR) [5]. During tumourigenesis, cancer cells often experience elevated levels of cellular stress, and they rely heavily on the UPR to adapt and survive in a hostile microenvironment [6]. The UPR consists of three main effector pathways that are initiated by ER-localised transmembrane proteins, namely inositol requiring enzyme 1α (IRE1α), protein kinase RNA like endoplasmic reticulum kinase (PERK) and activating transcription factor 6α (ATF6α) [7, 8].

More specifically, PERK is a type I transmembrane protein in the ER with a stress-sensing luminal domain and a cytosolic kinase domain. Under normal conditions, the ER chaperone GRP78/BiP binds to the luminal domain, preventing PERK activation. When unfolded proteins accumulate in the ER, GRP78/BiP dissociates from PERK, leading to its activation [9, 10]. Once activated, PERK phosphorylates eukaryotic initiation factor 2α (eIF2α) at serine-51, thereby disrupting ribosome assembly and subsequently reducing protein translation and synthesis. This decrease in protein synthesis, along with activation of signalling pathways (e.g. PI3K-Akt, MAPK, TRAF2, ASK1 and calcium signalling) associated with cell survival and proliferation, helps cancer cells adapt to the stressful tumour microenvironment [11, 12].

Despite PERK’s well-characterised role in the ER stress response, significant gaps remain in understanding of its mechanistic involvement, downstream signalling pathways and regulatory networks, particularly in the context of cancer [13, 14]. Emerging evidence suggests that *EIF2AK3* expression and function may be more context-dependent than previously recognised, warranting deeper investigation into its broader roles in cancer biology [15, 16].

Given its crucial role in cancer cell survival and proliferation, PERK is considered a potential therapeutic target to overcome drug resistance and improve cancer therapy outcomes [17, 18]. Further research is needed to fully understand the mechanisms by which PERK influences tumorigenesis and cancer progression.

Therefore, in this study, it was hypothesised that changes in *EIF2AK3* (or PERK) expression across tumour and normal tissues were associated with certain signalling pathways that play crucial roles in promoting cancer cell survival and proliferation. To address this, we investigated mRNA and protein expression of *EIF2AK3/*PERK across a range of normal and tumour tissues, using the Cancer Genome Atlas (TCGA), Genotype-Tissue Expression (GTEx) and Proteomics DB databases. Furthermore, mutation analysis was performed to determine whether specific point mutations may influence gene expression regulation. To explore the molecular context of *EIF2AK3* dysregulation, we conducted differential gene expression and pathway enrichment analyses (KEGG, GO and Reactome) to identify biological pathways associated with high or low *EIF2AK3* expression. Additionally, hierarchical clustering analysis was performed to uncover gene modules co-expressed with *EIF2AK3* across tumour types. Finally, we examined the association of *EIF2AK3* expression with ages, genders, tumour stages and survival endpoints in tumour patients.

## Methods

### Gene, protein and clinical datasets

RNA-seq data for primary tumours (*n* = 7,251) and their matched normal adjacent tissues (NATs) (*n* = 667) from the TCGA database, along with additional normal tissue samples from the GTEx project (*n* = 1,736) obtained from the OncoDB (https://oncodb.org/, accessed on 22 Jan 2026). Transcript expression levels were reported as Transcripts Per Million (TPM).

Relative expression as Log_2_ Fold Change (Log_2_ FC) for tumour samples (A) and control samples (B) was computed using the formula: Log_2_ (A/B) = Log_2_ (A) - Log_2_ (B), following the recommended approach for FC calculations provided by the UCSC Xena Browser (https://ucsc-xena.gitbook.io/project/faq/advanced-data-and-datasets#how-do-i-calculate-fold-change-fc, accessed on 17 Jul 2024). Batch correction was performed using the “limma::removeBatchEffect” function (v3.52.0). Comparisons of *EIF2AK3* expression were made between tumour tissues and NATs from the TCGA or normal tissues from GTEx in cases where NATs were unavailable.

Protein expression of PERK across various normal tissues was analysed using the ProteomicsDB database (https://www.proteomicsdb.org/, accessed on 15 Oct 2024). ProteomicsDB provides quantitative proteomics data generated from liquid chromatography-tandem mass spectrometry (LC-MS/MS) experiments across a broad spectrum of tissue types [19].

Further exploration of PERK protein abundance across normal tissues was conducted using the PaxDb database (Protein Abundances Across Organisms) (https://pax-db.org/, accessed on 15 Oct 2024). PaxDb compiles protein abundance data from a diverse range of tissues, providing both relative abundance values and rankings of PERK within the available proteome datasets [20].

Relative protein expression data for tumours (*n* = 680) and normal tissues (*n* = 315) of seven available primary tumour types (GBM, HNSC, LUAD, LUSC, PAAD, READ and UCEC), from the Clinical Proteomic Tumour Analysis Consortium (CPTAC) database were obtained from the Human Protein Atlas (https://www.proteinatlas.org/, accessed on 29 Oct 2024) [21]. Protein expression data for other tumour types were not available. The Human Protein Atlas database is generated from mass spectrometry-based proteomics data quantified using TMT-10 and TMT-11 labelling. Protein measurements were mapped to Ensembl v109 to ensure consistent gene annotation and normalised for relative protein expression (nRPX).

The normalised data for the patents’ ages, genders and American Joint Committee on Cancer (AJCC) pathologic tumour stages were obtained from the TCGA_PANCAN cohort through UCSC Xena browser (https://xena.ucsc.edu/, accessed on 22 Jan 2026). Gene expression values corresponded to Illumina HiSeq Pan-Cancer normalised RSEM data. Samples were filtered using the Xena phenotype categories age at diagnosis and AJCC pathological/clinical stage. Tumour samples were categorised with the cut() function into the following age groups: 0–29 years, 30–39 years, 40–49 years, 50–59 years, 60–69 years, 70–79 years and 80–100 years. Gender was classified as male or female, and pathological stages were grouped as Stage I to IV.

The public 24Q2 score dataset for cancer cell lines was obtained from the Cancer Dependency Map (DepMap) portal (https://depmap.org/portal/, accessed on 18 Jul 2024). This dataset includes the probability of dependency scores from CRISPR screen following *EIF2AK3* knockout for 1,179 tumour cell lines derived from 32 primary tumour lineages [22].

Coding region (CR) mutations (missense, multiple, splice and truncating mutations) and somatic copy number alteration (SCNA) types (amplification, deep deletion, diploid, shallow gain and deletion, gain) for primary tumour samples were obtained from cBioPortal using the “Query by Gene” function (https://www.cbioportal.org/, accessed on 21 Dec 2024), and downloaded from the “Plots” option. Data were filtered by mutation and SCNA types. *EIF2AK3* expression was derived from the RSEM using the batch normalised from Illumina HiSeq_RNASeqV2 pipeline, and sequence quality control, alignment and quantification followed standard procedures using FastQC, STAR and RSEM from cBioPortal [23, 24]. No additional filtering or harmonisation procedures were applied. Mutation annotations were standardised by the Genome Nexus.

Due to the absence of samples from cBioPortal, non-coding region (NCR) mutation data were retrieved from TCGA via the Genomic Data Commons (GDC) Data Portal (https://portal.gdc.cancer.gov/; accessed on 21 Dec 2024). Within the “Mutation Frequency” tool, *EIF2AK3* was specified as the gene of interest, and samples harbouring NCR mutations (5′UTR, 3′UTR and intron) were used to construct the mutation-positive cohort. The TCGA sample identifiers were extracted and matched to previously downloaded CR mutation and SCNA datasets obtained from cBioPortal. No additional filtering or harmonisation procedures were applied.

### Statistical analysis of differential *EIF2AK3* (PERK) expression between tumour tissues and corresponding control tissues

The relative *EIF2AK3* expression between tumour and control groups was analysed using two-tailed Welch’s t-tests using the t.test() function in R (v4.1.1), with *P*-values adjusted with the Benjamini-Hochberg method using the p.adjust(method = “BH”) function. Normalised relative protein expression (nRPX) was compared between tumour and normal samples using the same statistical framework. Boxplots were used to visualise expression patterns across tumour and normal samples using the ggplot2 package.

### Association between *EIF2AK3* expression and mutation status

Tumour samples from 28 primary tumour types (*n* = 10,073) were included in this study. The Kolmogorov-Smirnov test was first applied to assess the normality of the datasets, using the ks.test() function. All data were non-normally distributed, therefore Kruskal-Wallis tests (kruskal.test()) with Dunn’s multiple comparisons (dunnTest()) were used, to compare the difference in *EIF2AK3* expression between samples with different mutation or SCNA types. Furthermore, chi-square (χ²) test was applied to the datasets, using the chisq.test() function to check for overrepresentation.

### Association of *EIF2AK3* expression with patients’ ages, genders and tumour stages

To examine the association of *EIF2AK3* expression with age, gender and AJCC pathological stage, Log_2_ (RSEM + 1) values of 19 primary tumour types (*n* = 4,474) were analysed. The normality of the data was first checked by Shapiro-Wilk normality test using the shapiro.test() function. For age and tumour stage comparisons, normally distributed data were analysed using one-way analysis of variance (ANOVA) using the aov() function, followed by Tukey’s HSD post-hoc test (TukeyHSD()) for pairwise comparisons. Non-normally distributed data were assessed using the Kruskal-Wallis test (Kruskal.test()), with Dunn’s multiple comparisons test performed using the dunnTest() function. Gender-based comparisons were conducted using the Welch’s t-test (t.test()).

To evaluate the association of *EIF2AK3* expression with ages, genders and tumour stages, an analysis of covariance (ANCOVA) model was fitted using the lm() function. The model specified age as a continuous covariate, and gender and tumour stage as categorical factors. Interaction terms were included in the full model (EIF2AK3 ~ Age + Gender + Tumour Stage + Age: Gender + Age: Tumour Stage + Gender: Tumour Stage + Age: Gender: Tumour Stage). Type II sums of squares were computed using the Anova() function from the car package (v3.1-5), yielding *F-*statistics and corresponding *P*-values for each main effect and interaction term. The final results were reported as sums of squares, degrees of freedom, mean squares, *F*-statistics and *P*-values.

### Identification of differentially expressed genes (DEGs) between primary tumour samples with low and high *EIF2AK3* expression

Differentially expressed genes (DEGs) for 16 cancer types were analysed using the Gene Expression Profiling Interactive Analysis 2 (GEPIA2) platform (http://gepia2.cancer-pku.cn/#analysis/, accessed on 22 Jan 2026) using the “limma” function. *P*-values were adjusted by the Benjamini-Hochberg false discovery rate (FDR) method [25]. DEGs were defined as those with |log_2_ FC| > 1, and adjusted *P*-value **<** 0.05 [25].

### Functional enrichment analysis

Functional enrichment analysis was performed using the “clusterProfiler” R package (v4.1.1) [26]. Ensembl gene IDs were converted to Entrez IDs using the maplds() function in conjunction with the org.Hs.eg.db database (v3.14.0). Significantly enriched Kyoto Encyclopedia of Genes and Genomes (KEGG) pathways and Gene Ontology (GO) terms were identified using the enrichKEGG() and enrichGO() functions, respectively. GO terms were categorised into biological processes (BP), cellular components (CC) and molecular functions (MF). Significant pathways or GO terms were visualised based on gene count and adjusted *P* value < 0.05.

### Survival analysis

Survival analysis was conducted using data from 18 primary tumours (*n* = 7,462), using the GEPIA2 platform (http://gepia2.cancer-pku.cn/#analysis/, accessed on 02 Dec 2024). The samples were stratified into high and low *EIF2AK3* expression groups, which were defined as the lowest 25% and highest 25% of *EIF2AK3* expression levels. Overall survival (OS) and disease-free survival (DFS) were analysed using the log-rank test (Mantel-Cox), with the 95% confidence intervals applied to obtain Cox proportional hazards ratio. Kaplan-Meier plots were generated to illustrate the results [27].

### Hierarchical clustering, protein-protein interaction network and enrichment analysis

Top DEGs in primary tumours compared to normal tissue samples were retrieved from the GEPIA2 platform (http://gepia2.cancer-pku.cn/#analysis/, accessed on 20 Jul 2024). DEGs were processed and filtered in R (v4.1.1) using GEPIA2-derived tumour-versus-normal expression data across 20 primary tumour types. Genes were retained if they satisfied the significant expression changes of |log₂(RSEM + 1)| ≥ 1 and shared across primary tumours.

The filtered DEG list (*n* = 1,014) was mapped to gene symbols in the TCGA-GTEx pan-cancer expression database. To balance the number of tumour and normal samples, GTEx normal tissue data were incorporated. Due to discrepancies in identifiers and missing gene symbols, 919 genes were successfully mapped and used for further analysis.

Normalised expression data from 25 primary tumour types and their GTEx-matched normal tissues were used to compute tumour-versus-normal expression differences. Row-wise Z-score normalisation was applied to the expression matrix. Pairwise similarity between genes and tumour types was computed using a correlation-based distance metric (1 - correlation coefficient) with the cor() function. The resulting correlation matrix was converted to a distance object using as.dist(). Hierarchical clustering was performed using the hclust() function. Heatmaps were generated in R (v4.5.1) using the heatmap() function. Dendrogram structures derived from hierarchical clustering were applied through the Rowv and Colv parameters. Colour gradients represent relative tumour-versus-normal expression changes, with red indicating upregulation and blue indicating downregulation. The position of *EIF2AK3* was annotated. No further validation metrics (e.g., silhouette widths) were applied; clustering patterns were evaluated visually using the heatmaps.

To explore the functional relevance of *EIF2AK3*-associated gene clusters, protein-protein interaction (PPI) networks were constructed using the STRING database (v11.5; accessed on 11 May 2025). The full STRING network was applied, incorporating both functional and physical protein associations derived from all active interaction sources, including text mining, experimental data and curated databases. A minimum required interaction score of 0.700 (high confidence) was imposed. Network clustering was performed using k-means clustering (K = 4), and disconnected nodes were excluded from visualisation.

The resulting PPI network was imported into Cytoscape (v3.10.3) for graphical refinement. Edges between clusters were hidden to improve visual clarity of cluster-specific interaction patterns. The network was subsequently visualised.

Functional enrichment analysis was conducted within the STRING database for GO terms (BP, MF and CC), as well as disease association datasets. Statistical significance was determined using FDR correction, with adjusted *P*-values < 0.05 considered significant. For each category, the top 10 most significantly enriched terms based on FDR were selected for presentation.

## Results

### Differential regulation of *EIF2AK3* (PERK) gene and protein expression across malignancies

For a comprehensive evaluation of *EIF2AK3* expression in primary tumours, two different RNA-seq datasets from the TCGA and TARGET cohorts were used in our analysis, alongside corresponding NATs or normal tissues from the GTEx database. 28 TCGA primary tumours types were included in the analysis, encompassing adrenocortical carcinoma (ACC), bladder urothelial carcinoma (BLCA), breast invasive carcinoma (BRCA), cervical squamous cell carcinoma and endocervical adenocarcinoma (CESC), cholangiocarcinoma (CHOL), colon adenocarcinoma (COAD), diffuse large B-cell lymphoma (DLBC), oesophageal carcinoma (ESCA), glioblastoma multiforme (GBM), head and neck squamous cell carcinoma (HNSC), kidney chromophobe (KICH), kidney clear cell carcinoma (KIRC), lower-grade glioma (LGG), kidney papillary cell carcinoma (KIRP), liver hepatocellular carcinoma (LIHC), lung adenocarcinoma (LUAD), lung squamous cell carcinoma (LUSC), ovarian serous cystadenocarcinoma (OV), pancreatic adenocarcinoma (PAAD), pheochromocytoma and paraganglioma (PCPG), prostate adenocarcinoma (PRAD), rectal adenocarcinoma (READ), skin cutaneous melanoma (SKCM), stomach adenocarcinoma (STAD), testicular germ cell tumour (TGCT), thyroid carcinoma (THCA), thymoma (THYM) and uterine corpus endometrioid carcinoma (UCEC).

**Fig. 1:**
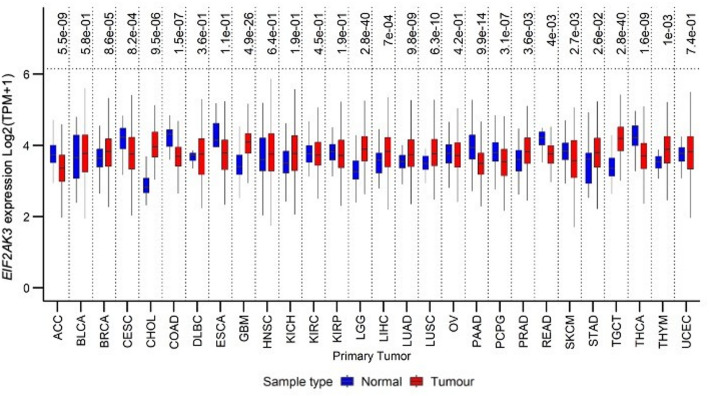
*EIF2AK3* gene expression levels in primary tumours and corresponding normal tissues across 28 TCGA primary tumour types. RNA-seq data were Log₂ (RSEM + 1)-transformed. Expression levels are presented as median ± interquartile range (IQR) in box plots, with blue representing normal tissues and red representing tumours. *P*-values from Welch’s t-test comparisons between tumour and normal tissues are indicated above each plot and were adjusted for multiple comparisons using the Benjamini-Hochberg (BH) method

A significant upregulation of *EIF2AK3* mRNA expression was observed in 12 of the 28 tumour types when compared to their corresponding NATs or healthy tissues. The tumour types included LGG (Log_2_FC = 1.46, *P* = 2.8e-40), GBM (Log_2_FC = 1.40, *P* = 4.9e-26), CHOL (Log_2_FC = 1.78, *P* = 9.5e-06) and TGCT (Log_2_FC = 1.49, *P* = 2.8 e-40) (Fig. 1).

Conversely, significant downregulation of *EIF2AK3* expression was identified in seven primary tumour types when compared to their respective NATs or normal tissues. These tumour types included ACC (Log_2_FC = -0.69, *P* = 5.5e-09), CESC (Log_2_FC = -0.82, *P* = 8.2e-04), COAD (Log_2_FC = -0.72, *P* = 1.5e-07), PAAD (Log_2_FC = -0.69, *P* = 9.9e-14), PCPG (Log_2_FC = -0.80, *P* = 3.1e-07), READ (Log_2_FC = -0.77, *P* = 4e-03) and THCA (Log_2_FC = -0.58, *P* = 1.6e-9) (Fig. 1).

Given the differential expression of *EIF2AK3* across different tumour types observed in the transcriptomic analysis, it was important to investigate whether these changes reflect in corresponding protein expression levels. This is particularly relevant because PERK protein undergoes post-translational modifications, such as phosphorylation, to fulfill its functional roles [28].

The protein expression data from normal tissues were first obtained from ProteomicsDB and compared. The results revealed detectable levels of full-length PERK protein expression in various normal tissues, including the salivary gland, pancreas, thyroid gland and bone marrow (Supplementary Fig. S1).

**Fig. 2:**
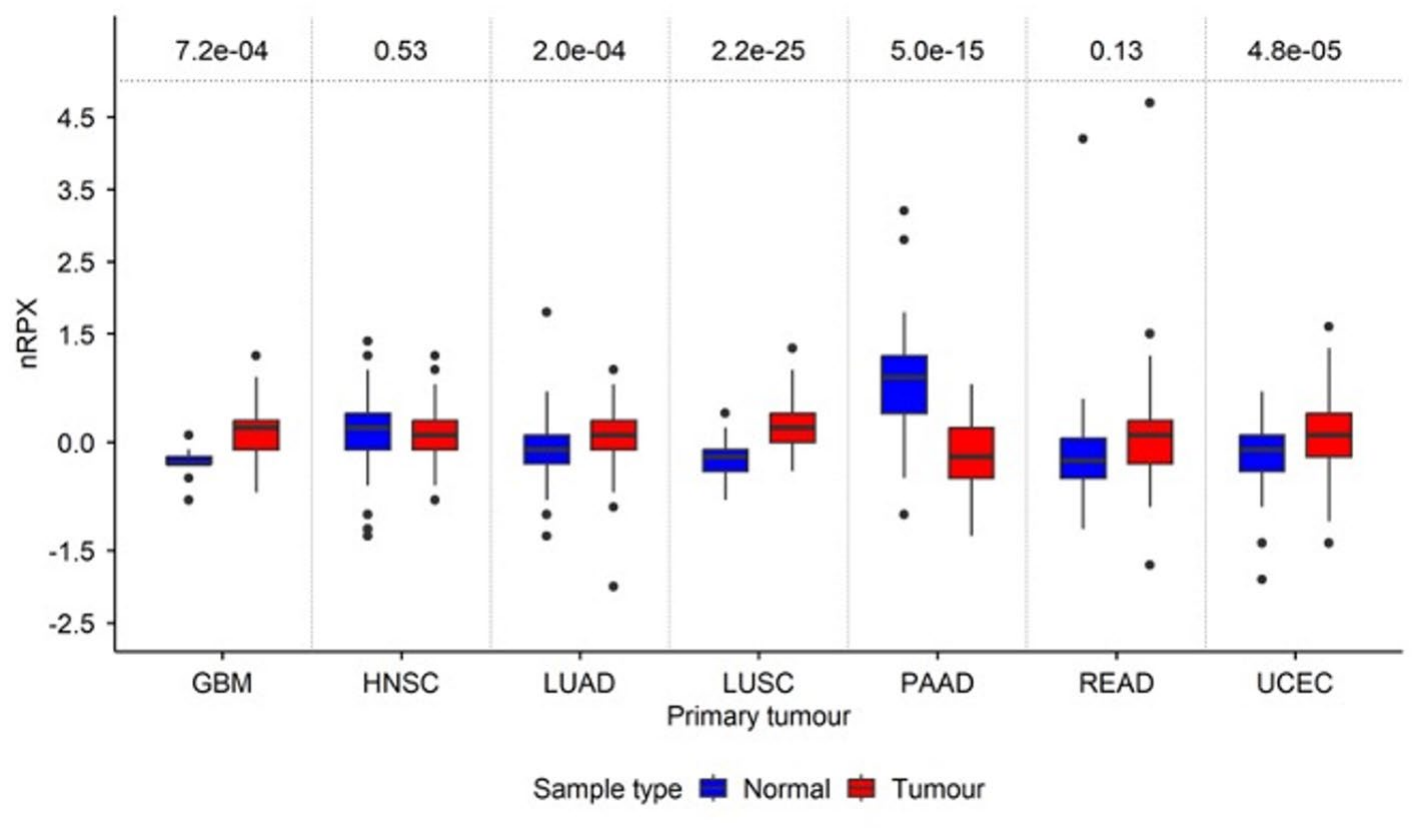
Relative protein expression of PERK across seven primary tumour types compared with corresponding normal tissues. Relative protein expression was normalised relative to control tissues (nRPX values) and Log₂-transformed. Data are presented as median ± interquartile range (IQR) in box plots, with blue representing normal tissues and red representing tumours. *P*-values from Welch’s t-test comparisons between tumour and normal samples are shown above each plot and were adjusted for multiple testing using the Benjamini-Hochberg (BH) correction

To broaden the scope of the expression analysis and link the observations from normal tissue expression in human to disease contexts, nRPX values were examined across seven available primary tumour types, with relative protein expression levels calculated by comparing with normal tissue samples. The analysis included UCEC, READ, PAAD, LUSC, LUAD, HNSC and GBM. The results revealed significant upregulation of protein expression in the primary tumour types including GBM (Log_2_FC = 1.17, *P* = 7.2e-04), LUSC (Log_2_FC = 1.21, *P* = 2.2e-25), LUAD (Log_2_FC = 1.11, *P* = 2.0e-04) and UCEC (Log_2_FC = 1.04, *P* = 4.8e-05). In contrast, significant downregulation was detected in PAAD (Log_2_FC = -0.59, *P* = 5.0e-15), and no significant differences in protein expression were observed in READ (Log_2_FC = 1.09, *P* = 0.13) or HNSC (Log_2_FC = 0.105, *P* = 0.5) (Fig. 2).

### Cancer cell line transcriptomics and proteomics analysis

The analysis of *EIF2AK3* expression across cancer cell lines from DepMap provides a comprehensive view of its role in different cancer contexts. Dependency score analysis of 1,179 cancer cell lines across 31 lineages revealed that majority of the cell lines did not show significant reliance on *EIF2AK3* for survival, except for 14 cell lines (NZM7, SKMM2, RPMI 2650, PEER, P2URK562, UPCISCC 131, ECC4, NCCES1CI, WCIH446, BL70, HEC251, KN562, HAP1 and SKMEL9), which showed dependency scores lower than − 0.5, indicating that *EIF2AK3* knockout generally did not affect the viability of most of the cancer cell lines. This suggests a limited role for *EIF2AK3* in maintaining cell viability under controlled experimental conditions (Supplementary Fig. S2).

To gain deeper insights into protein expression, proteomic data were retrieved from the ProteomicsDB database, which included MS1 intensity-derived iBAQ scores to quantify PERK protein abundance across a wide array of cancer cell lines [29]. This analysis included tumour cell lines such as BRCA (MCF-7, MDA-MB-453, HCC-1937, MFM-223, HMT-3522), COAD (HT-29, SW-403, SW-948, LIM1863), OV (CaR-1), LCLs (Jurkat), OS (U2-OS), CESC (HeLa), PAAD (Colo-678), CRMA (NCI-H716), GBM (GaMG), LUAD (A-549), PRAD (LNCaP), HEK (HEK-293) and MECs (HMEpC).

The findings revealed substantial variability in PERK protein expression across tumour cell lines. The highest levels of PERK protein expression were observed in OS (U2-OS), CESC (HeLa), BRCA (MDA-MB-453, MCF-7), COAD (SW-948, SW-403), CRMA (NCI-H716), OV (CaR-1), PAAD (Colo-678) and GBM (GaMG). In contrast, relatively lower levels of PERK expression were detected in LUAD (A-549), PRAD (LNCaP), HEK (HEK-293) and LCLs (Jurkat) (Supplementary Fig. S3).

### Effect of point mutations on *EIF2AK3* expression

**Fig. 3:**
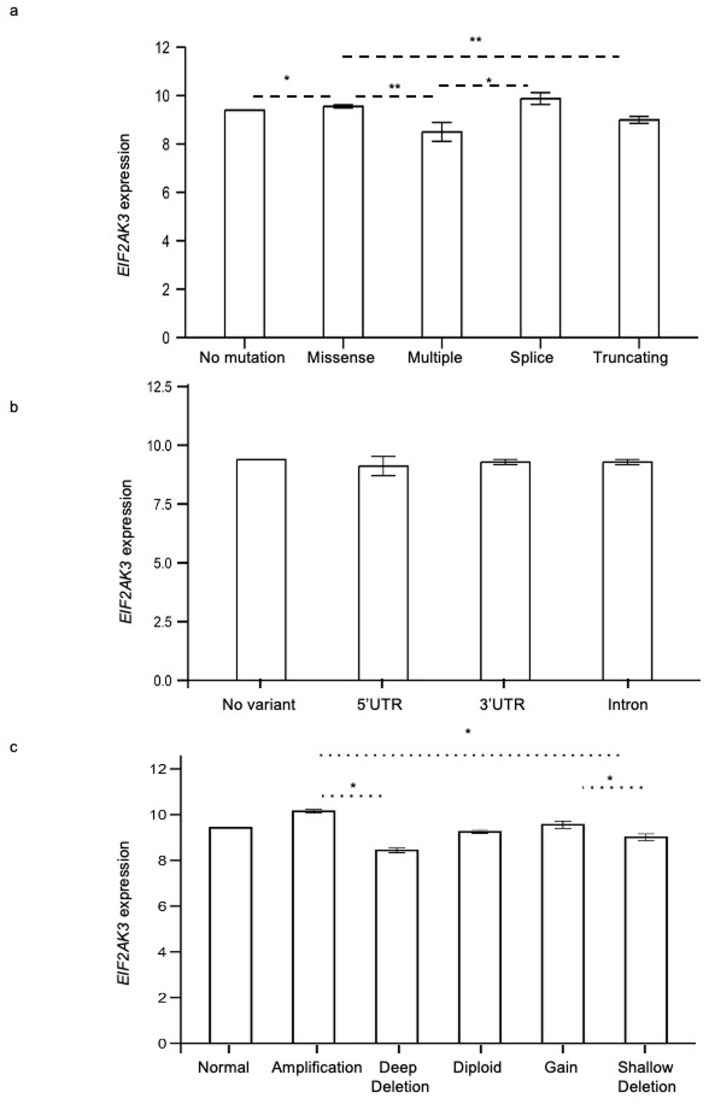
*EIF2AK3* expression compared among patient samples with (**a**) coding region (CR) mutations, (**b**) non-coding region (NCR) mutations, and (**c**) somatic copy number alterations (SCNA). RNA-seq expression data (Log₂ [RSEM + 1]) are shown as mean ± SEM. Statistical analysis was performed using Kruskal-Wallis tests with *P*-values adjusted for multiple comparisons using Dunn’s test. *P < 0.05 and **P < 0.01

Tumour samples from 28 primary tumour types (ACC, AML, BRCA, ESCA, CESC, COAD, BLCA, CHOL, GBM, HNSC, LUAD, LUSC, LIHC, PAAD, PRAD, STAD, SKCM, READ, OV, KICH, KIRP, LGG, ENSC, UCEC, THCA, THYM, OM, PHEO and PGL; *n* = 10,072) were used to investigate association between *EIF2AK3* expression and mutations within the CRs and SCNA within the *EIF2AK3* gene locus (Fig. 3). The majority of (98.27%) tumour samples (9,898 out of 10,072) within the *EIF2AK3* gene locus. Significant effect of CR mutations on *EIF2AK3* expression was observed (*P* = 6.5e-05). Samples with missense mutation exhibited significantly higher *EIF2AK3* expression (Log₂FC = 0.159, *P* = 0.017) compared to those with no mutations, truncating mutations (Log₂FC = -0.400, *P* = 0.0013) and multiple mutations (Log₂FC = -0.899, *P* = 0.0075. Similarly, samples with splice mutation exhibited significantly higher *EIF2AK3* expression compared to those with multiple mutations (Log₂FC = 0.479, *P* = 0.026) (Fig. 3a). A chi-squared test revealed that there was significant overrepresentation of CR mutations between high and low *EIF2AK3* expression groups (X^2^(4) = 13.13, *P* = 0.0106). 0.94% of samples in the high *EIF2AK3* group had CR mutation (95 out of 10,072) compared to 0.78% samples in the low *EIF2AK3* group (78 out of 10,072) (Supplementary Table 1a).

For NCR mutations, 2.07% of tumour samples (208 out of 10,072) had no identified mutations within the *EIF2AK3* gene locus (Fig. 3b). Comparison of high and low *EIF2AK3* groups resulted in no significance (*P* = 0.75), and no overrepresentation of *EIF2AK3* expression (X^2^ (3) = 4.27, *P* = 0.96) was observed in samples with high and low *EIF2AK3* expression by a chi-square test. 1.04% of tumour samples in the high *EIF2AK3* group (105 out of 10,072) had NCR mutations compared to 1.02% (103 out of 10,072) in the low *EIF2AK3* group (Supplementary Table 1b).

Regarding SCNAs, 98.53% of tumour sample (9,924 out of 10,072) were found to have no identified SCNA mutations. There was a significant effect of SCNA mutations on *EIF2AK3* expression (*P* = 0.001). None of the mutation types had significant difference in *EIF2AK3* expression compared to normal copy number. However, samples with amplification mutation had significantly higher *EIF2AK3* expression (Log_2_FC = 1.08, *P* = 0.024) compared to the deep deletion mutation. Samples with gain mutation also had significantly higher *EIF2AK3* expression (Log_2_FC = 0.15, *P* = 0.05) compared to samples with shallow deletion mutation. There was also a significantly higher *EIF2AK3* expression in samples with amplification (Log_2_FC = 0.16, *P* = 0.0435) compared to those with shallow deletion mutation (Fig. 3c). A chi-square test showed no overrepresentation in high and low *EIF2AK3* groups for any SCNA mutation types (X2 (5) = 12.92, *P* = 0.057), with 0.84% (85 out of 10,072) of samples in the high *EIF2AK3* group had SCNA mutations compared to 0.85% (86 out of 10,072) in the low *EIF2AK3* group (Supplementary Table 1c).

### Association between *EIF2AK3* expression and patients’ ages, genders and tumour stages

**Fig. 4:**
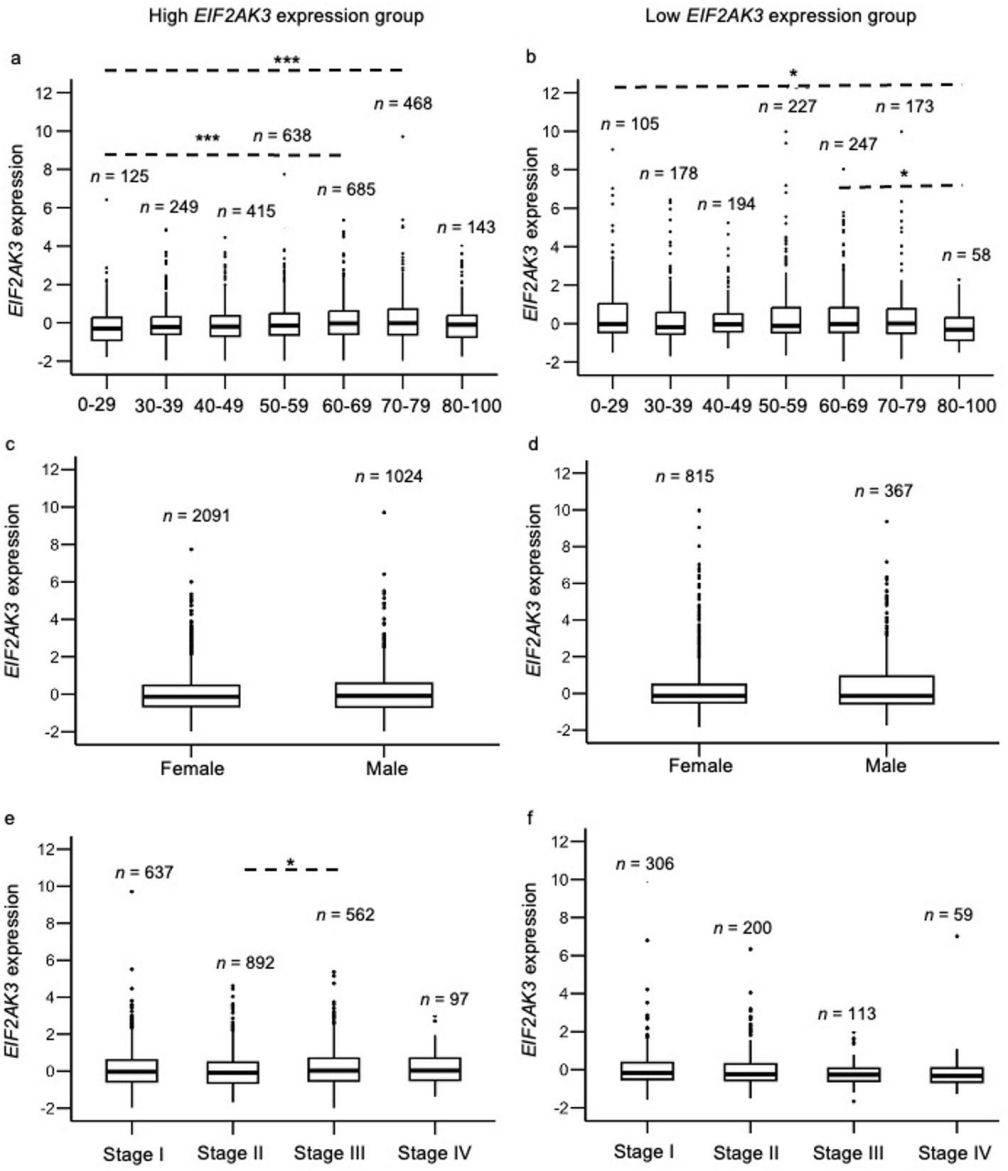
*EIF2AK3* gene expression comparisons across cohorts stratified by (**a**, **b**) age, (**c**, **d**) gender and (**e**, **f**) pathological stage, in groups with high and low *EIF2AK3* expression. Statistical analysis was performed using Kruskal-Wallis tests followed by Dunn’s multiple comparisons test. Data are presented as mean ± SEM, with sample sizes (*n*) indicated on the plots. *P < 0.05 and *** *P* < 0.001

The association between *EIF2AK3* expression and patients’ demographics, including ages, genders, and AJCC pathological stages at initial diagnosis, was investigated. RNA-seq data were analysed across two primary tumour cohorts: one consisting of tumour types with high *EIF2AK3* expression (BRCA, CHOL, GBM, LGG, LIHC, LUSC, LUAD, PRAD, SKCM, STAD, TGCT and THYM) and another with low *EIF2AK3* expression (ACC, COAD, READ, PAAD, PCPG and THCA). Tumour samples with high *EIF2AK3* expression were significantly associated with patients’ ages (*P* = 0.000686). Notably, *EIF2AK3* expression was significantly higher in the 70–79 age group (Log_2_FC = 0.59, *P* = 0.0037) compared to the 20–29 age group. Similarly, the 60–69 age group also exhibited significantly higher *EIF2AK3* expression compared to the 20–29 age group (Log_2_FC = 0.57, *P* = 0.00623) (Fig. 4a).

On the other hand, tumours with low *EIF2AK3* expression were also significantly associated with patients’ ages (*P* = 0.047). Specifically, the 80–100 age group showed significantly lower *EIF2AK3* expression compared to the 0–29 (Log_2_FC = -0.43, *P* = 0.05) and 60–69 (Log_2_FC = -0.62, *P* = 0.0038) age groups (Fig. 4b).

No significant differences in *EIF2AK3* expression were observed between females and males in tumour types with high *EIF2AK3* expression (*P* = 0.84) and low *EIF2AK3* expression (*P* = 0.13) (Fig. 4c, d). However, when examining AJCC pathological stages, a significant association was observed in tumours with high *EIF2AK3* expression (*P* = 0.032). Patients in Stage III exhibited significantly higher *EIF2AK3* expression (Log_2_FC = 0.19, *P* = 0.035) compared to those in Stage II (Fig. 4e). No significance was observed in any stages of the low *EIF2AK3* group (*P* = 0.12) (Fig. 4f).

Individual tumour types were examined for age, gender and AJCC pathological stages’ correlations with *EIF2AK3* expression. No significant associations were found between *EIF2AK3* expression and either age or gender across any tumour types (Supplementary Fig. S4 and S5).

In STAD, patients with Stage I exhibited significantly higher *EIF2AK3* expression compared to those with Stage IV (Log_2_FC = 1.13, *P* = 0.0036) and Stage II (Log_2_FC = 0.79, *P* = 0.017). In SKCM, patients with Stage II exhibited significantly higher *EIF2AK3* expression (Log_2_FC = 0.74, *P* = 0.053) than Stage IV. In TGCT, patients with Stage I exhibited significantly higher *EIF2AK3* expression (Log_2_FC = 1.54, *P* = 0.0048) compared to Stage III, and patients with Stage II also exhibited higher *EIF2AK3* expression (Log_2_FC = 1.3, *P* = 0.017) than Stage III (Supplementary Fig. S6).

To assess whether the association of *EIF2AK3* expression with age, gender and tumour stage, ANCOVA was performed. *EIF2AK3* expression was specified as the dependent variable, ages as a continuous predictor, and genders and tumour stages as covariates. Only tumour samples with complete data for *EIF2AK3* expression, age, gender and tumour stage were included in the analysis.

In tumours with high *EIF2AK3* expression (LIHC, LUAD, LUSC, STAD, SKCM and TGCT), *EIF2AK3* levels were significantly associated with age (*P* = 0.003), gender (*P* = 0.0003) and tumour stage (*P* = 0.035). A statistically significant interaction effect was observed between gender and tumour stage (*P* = 0.037). No significant interaction effects were detected for age × tumour stage (*P* = 0.160), age × gender (*P* = 0.483), or age × gender × tumour stage (*P* = 0.106) (Supplematary Table 2a).

In tumours with low *EIF2AK3* expression (PAAD, READ and THCA), *EIF2AK3* levels were not significantly associated with age, tumour stage, or gender after adjustment for covariates and interaction terms (all *P* > 0.05). Specifically, no significant main effects were detected for age (*P* = 0.72), tumour stage (*P* = 0.33), or gender (*P* = 0.93). Consistently, interaction analyses revealed no significant effects for age × tumour stage (*P* = 0.15), age × gender (*P* = 0.09), gender × tumour stage (*P* = 0.58), or age × gender × tumour stage (*P* = 0.79) (Supplementary Table 2b).

### Association of *EIF2AK3* expression in primary tumours with specific transcriptomic alterations

**Fig. 5:**
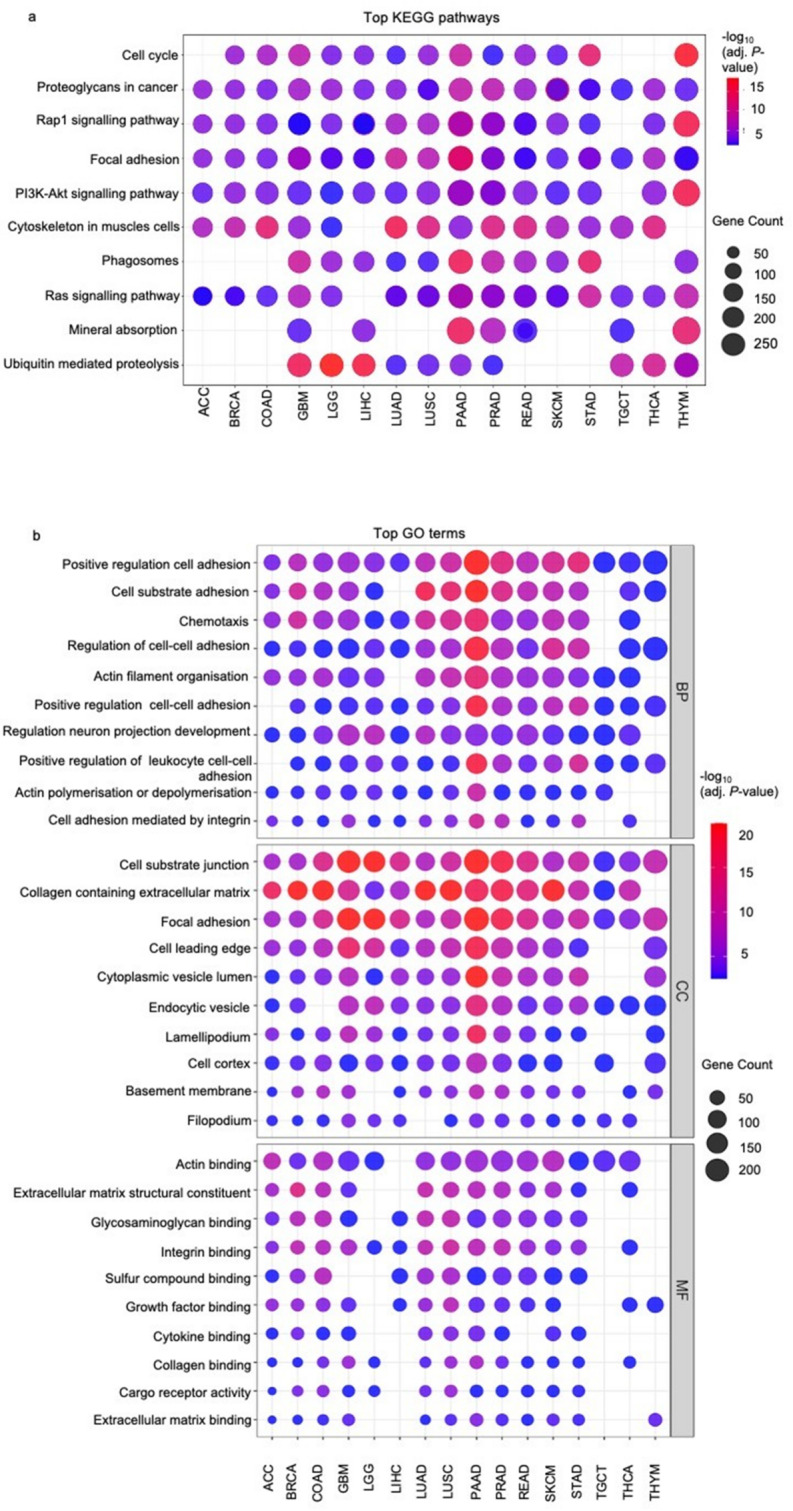
**a** KEGG pathway and **b** GO enrichment analyses of the differentially expressed genes in all primary tumour samples. BP: biological process; CC: cellular component; MF: molecular function. Each bubble represents a pathway or GO term enriched in a specific cancer type. Data are represented as -log_10_(adj. *P*-value). Bubble size indicates the number of genes associated with each term, while bubble colour reflects the magnitude of -log_10_(adj. *P*-value)

KEGG pathway analysis revealed significant enrichment of multiple cancer-related pathways across tumour types. Among the top enriched pathways, the cell cycle pathway was significantly enriched in 13 tumour types, while proteoglycans in cancer and focal adhesion pathways were consistently enriched across all 16 tumour types. Other commonly enriched pathways included Rap1 signalling and PI3K-Akt signalling (15 tumour types each), cytoskeleton regulation in muscle cells (14 tumour types), and phagosome formation (11 tumour types). Additional cancer-relevant pathways such as Ras signalling (15 tumour types), mineral absorption (7 tumour types), and ubiquitin-mediated proteolysis (10 tumour types) were also significantly enriched (Fig. 5a).

Tumours with high *EIF2AK3* expression (BRCA, LGG, GBM, LIHC, LUSC, LUAD, PRAD, SKCM, STAD, and THYM) exhibited strong enrichment in pathways related to cell cycle, Rap1 signalling, PI3K-Akt signalling (10 tumour types each), and proteoglycans in cancer (all tumour types), alongside cytoskeleton in muscle cells (9 tumour types) and ubiquitin-mediated proteolysis (8 tumour types). Additional enriched pathways included nucleocytoplasmic transport (6 tumour types), phagosomes (9 tumour types), and neurodegenerative disease-related pathways such as amyotrophic lateral sclerosis and prion disease (5 tumour types each) (Supplementary Fig. S7a).

In contrast, tumours with low *EIF2AK3* expression (ACC, COAD, PAAD, READ, and THCA) demonstrated consistent enrichment in focal adhesion, Ras signalling, Rap1 signalling, cytoskeleton regulation in muscle cells, and AGE-RAGE signalling in diabetic complications across all five tumour types. Other significantly enriched pathways included cell cycle, chemical carcinogenesis, reactive oxygen species, and pathogenic *Escherichia coli* infection (3 tumour types each), as well as prion disease and ubiquitin-mediated proteolysis (4 tumour types) (Supplementary Fig. S7b).

The functional characteristics of DEGs from the combined datasets were first investigated using GO enrichment analysis and grouped into three major categories: BP, CC and MF. Several GO terms were significantly enriched across multiple cancer types. In the BP category, DEGs were strongly associated with “positive regulation of cell adhesion” (16 tumour types), “chemotaxis” (14 tumour types), and “actin filament organisation” (14 tumour types). Within the CC category, prominent enrichment was observed in “cell substrate junction” (16 tumour types), “collagen-containing extracellular matrix” (15 tumour types), “focal adhesion” (16 tumour types), and “cell leading edge” and “cytoplasmic vesicle lumen” (14 tumour types). In the MF category, terms such as “actin binding” (14 tumour types), “extracellular matrix structural constituent” (12 tumour types), “glycosaminoglycan binding” (12 tumour types), “integrin binding” (14 tumour types), “sulfur compound binding” (11 tumour types), and “growth factor binding” (13 tumour types) were among the most significantly enriched (Fig. 5b).

In tumours with high *EIF2AK3* expression, GO analysis revealed significant enrichment of several biological processes and cellular structures across multiple cancer types. Within the BP category, enriched terms included “positive regulation of cell adhesion”, “negative regulation of protein modification”, and “positive regulation of cell-cell adhesion”, each observed in 11 tumour types. In the CC category, the most significantly enriched terms were “cell substrate junction” and “focal adhesion” (11 tumour types each). In the MF category, key enriched terms included “actin binding” (9 tumour types), “integrin binding” (9 tumour types), and “extracellular matrix structural constituent” (7 tumour types) (Supplementary Fig. S8a).

In contrast, tumours with low *EIF2AK3* expression showed enrichment in GO terms reflecting structural maintenance and adhesion-mediated signalling. In the BP category, top enriched terms included “cell-substrate adhesion,” “actin filament organisation”, “cell-matrix adhesion”, and “axonogenesis” across all five tumour types. CC terms such as “cell substrate junction”, “basal part of cell”, and “actin-based cell projection” were similarly enriched in all five tumour types. In the MF category, significant enrichment was observed for “actin binding” (5 tumour types), “glycosaminoglycan binding” (4 tumour types), “extracellular matrix structural constituent”, and “integrin binding” (5 tumour types) (Supplementary Fig. S8b).

### Correlation of *EIF2AK3* expression with survival endpoint in patients

The association between *EIF2AK3* expression levels and patients’ survival outcomes, including OS and DFS, was examined across 17 primary tumour types (ACC, BRCA, COAD, CHOL, LGG, GBM, LIHC, LUAD, LUSC, PRAD, SKCM, STAD, PAAD, READ, TGCT, THCA and THYM). Patients were stratified into two groups based on *EIF2AK3* expression: the top 25% (high expression) and the bottom 25% (low expression).

**Fig. 6:**
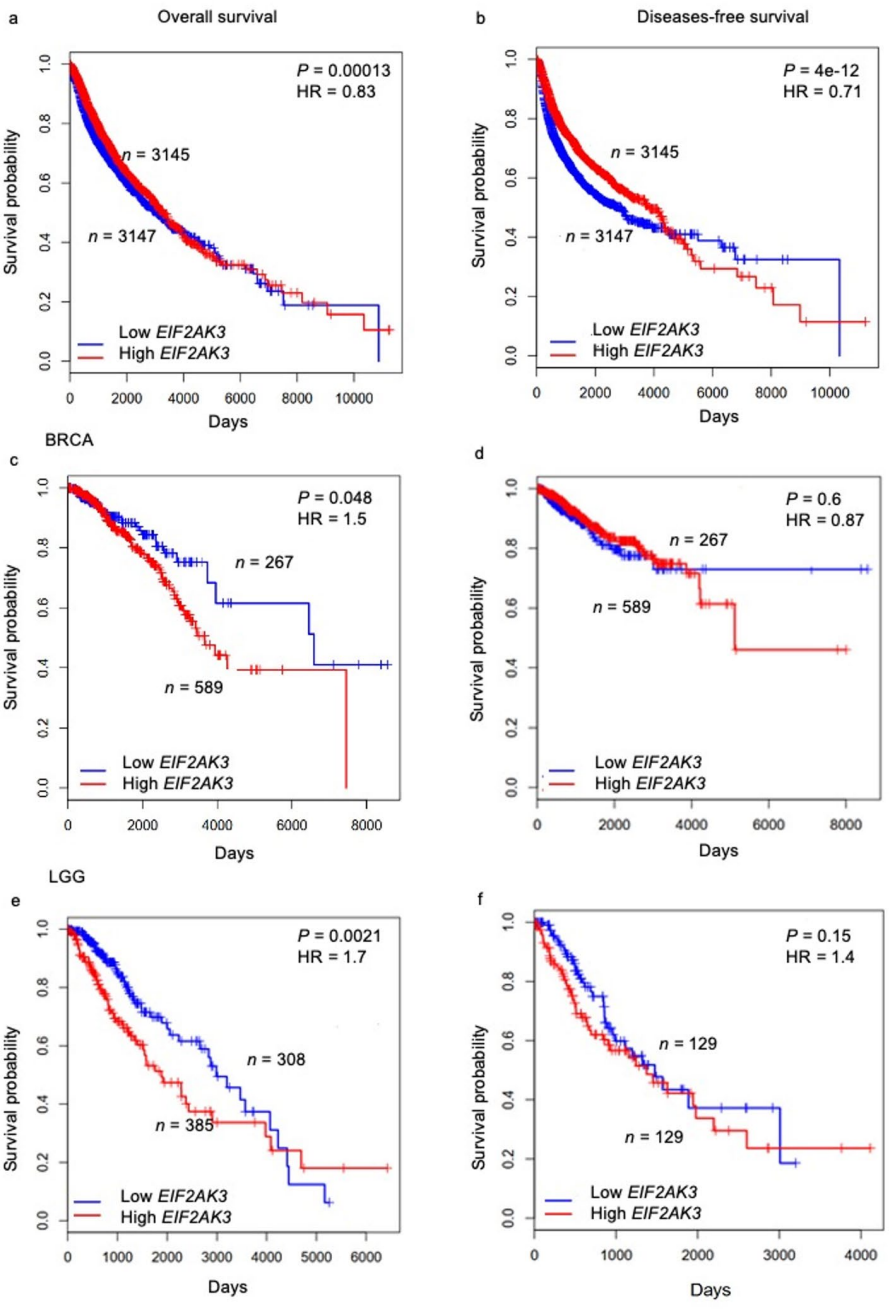
Kaplan-Meier survival analysis of (**a**) overall survival (OS) and (**b**) disease-free survival (DFS) stratified by *EIF2AK3* expression (high vs. low). Cancer-type specific analyses are shown for BRCA: (**c**) OS and (**d**) DFS; and LGG: (**e**) OS and (**f**) DFS. The x-axis denotes survival time (days) and the y-axis shows survival probability. Numbers at risk are displayed on the plots. *P*-values (log-rank test) and hazard ratios (HR) are indicated. Red lines represent high *EIF2AK3* expression; blue lines represent low *EIF2AK3* expression

Surprisingly, across all tumour types, high *EIF2AK3* expression was associated with improved OS (HR = 0.85, *P* = 0.00013), with a median survival of 11,021 days in the high *EIF2AK3* group compared to 10,800 days in the low *EIF2AK3* group (Fig. 6a). Similarly, high *EIF2AK3* expression was associated with improved DFS (HR = 0.71, *P* = 4e-12), with a median survival of 11,012 days in the high *EIF2AK3* group compared to 10,200 days in the low *EIF2AK3* group (Fig. 6b). This finding suggests potential context-specific roles of *EIF2AK3* across tumour types.

When investigating individual tumour types, BRCA patients with high *EIF2AK3* expression had significantly lower OS (HR = 1.5, *P* = 0.048), with a median survival of 7,854 days compared to 9,012 days in the low *EIF2AK3* group (Fig. 6c). LGG patients with high *EIF2AK3* expression exhibited a significantly lower OS (HR = 1.7, *P =* 0.0021), with a median OS of 4,012 days compared to 5,123 days in the low *EIF2AK3* group (Fig. 6d). There was no significant difference in DFS in any of primary tumour types, however (Supplementary Fig. S9).

### *EIF2AK3*-associated gene network and functional enrichment analysis

**Fig. 7:**
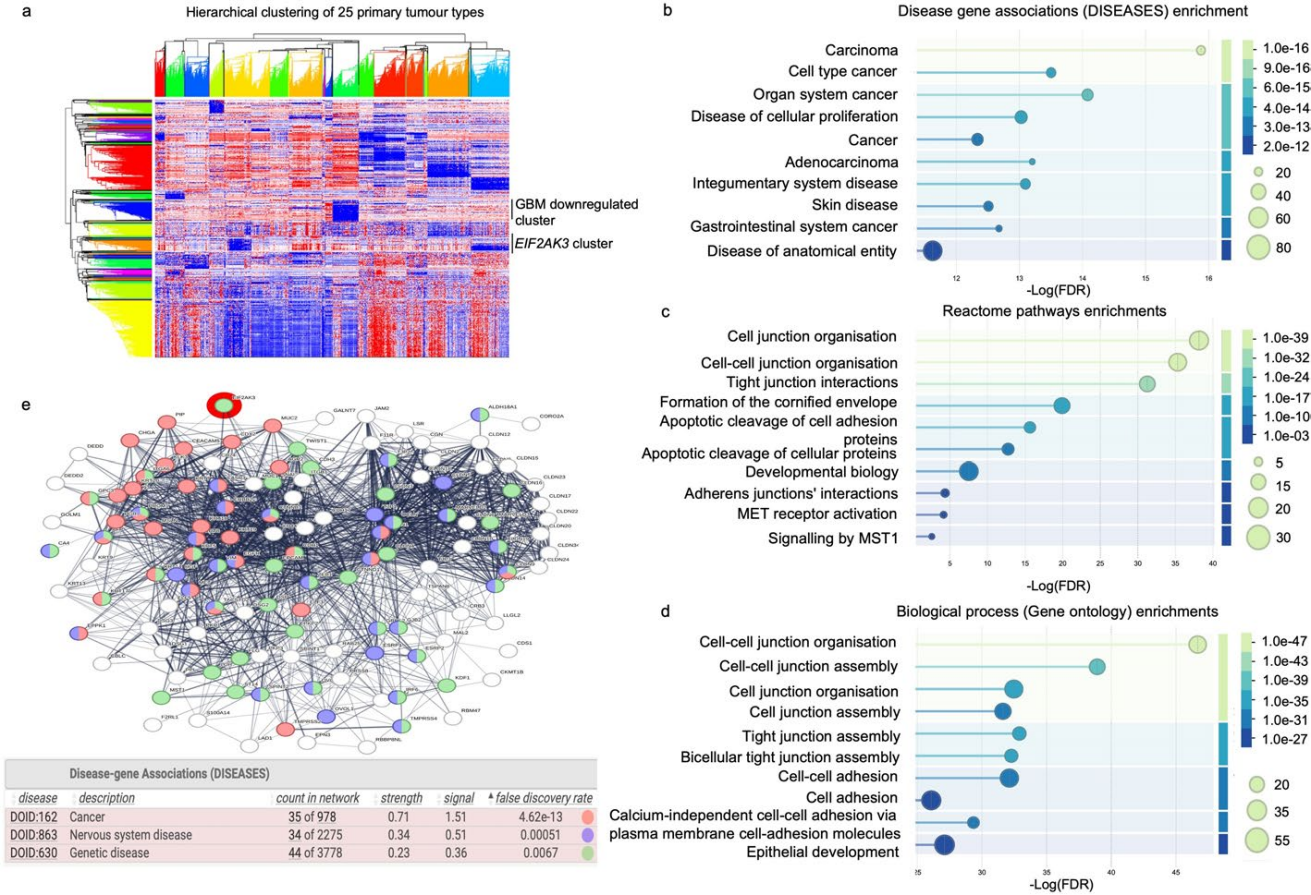
*EIF2AK3*-associated gene network and functional enrichment analysis in human cancers. **a** Hierarchical clustering of common DEGs from 25 primary tumour types in the TCGA dataset. A distinct *EIF2AK3* gene cluster was identified. Interestingly, *EIF2AK3* gene cluster was downregulated in GBM and LGG. **b** Disease gene association enrichment, **c** reactome pathway enrichment and **d** Gene Ontology (biological process) enrichment analysis of the *EIF2AK3* cluster identified from **a**. The x-axis demonstrates enrichment score (-Log (FDR)). Bubble size reflects the number of genes associated with each term, while bubble colour indicates statistical significance based on the -Log (FDR). **e** Protein-protein interaction (PPI) network of the *EIF2AK3* cluster constructed using the STRING database, showing its interaction partners and their disease associations

Hierarchical clustering analysis of shared DEGs across 25 primary tumour types revealed several gene clusters, including a distinct *EIF2AK3* gene cluster. Notably, while *EIF2AK3* expression was upregulated in GBM and LGG compared to normal samples, its associated gene cluster was downregulated (Fig. 7a).

Functional enrichment using the DISEASES database revealed significant overrepresentation of cancer-related categories, including carcinoma (FDR = 1.0e-16), organ system cancer (FDR = 4.0e-14) and diseases of cellular proliferation (FDR = 3.0e-13) (Fig. 7b).

Reactome pathway analysis identified cell junction organisation as the most significantly enriched pathway (FDR = 1.0e-40), followed by cell-cell junction organisation (FDR = 1.0e-35) and tight junction interactions (FDR = 1.0e-30), indicating a central role in intercellular adhesion and barrier function. Other pathways, including formation of the cornified envelope and apoptotic cleavage of adhesion proteins, MET receptor activation and signalling by MST1 were also enriched (Fig. 7c).

GO enrichment analysis supported these findings, highlighting strong enrichment in processes such as “cell-cell junction organisation” (FDR = 1.0e-47), “tight junction assembly” (FDR = 1.0e-35) and “cell-cell adhesion” (FDR = 1.0e-31) (Fig. 7d).

To further explore functional associations, we constructed a PPI network for the *EIF2AK3*-associated gene cluster, expanded to include 100 related proteins. This network revealed dense interconnectivity among genes, with many proteins showing direct or indirect associations. Disease enrichment analysis of the network showed strong links to cancer (35 genes; FDR = 4.62e-13), nervous system diseases (34 genes; FDR = 0.00051) and genetic disorders (44 genes; FDR = 0.0067), suggesting broad roles in tumour biology, neurodegeneration and heritable conditions (Fig. 7e).

## Discussion

Tumour cells frequently encounter stressful conditions such as hypoxia and nutrient deprivation, which trigger the accumulation of unfolded proteins in the ER, leading to ER stress [30]. This study focuses on *EIF2AK3* (PERK), a critical regulator of the UPR, and its association with tumour biology and cellular stress adaptation. Analysis of *EIF2AK3* mRNA and protein expression across diverse primary tumour types revealed significant transcriptional and translational variability, underscoring its context-dependent role in cancer.

*EIF2AK3* expression from tumour tissue samples compared to matched adjacent normal tissues or normal tissues revealed that *EIF2AK3* expression was significantly upregulated in 12 tumour types and downregulated in 7. Elevated *EIF2AK3* expression was observed in cancers such as GBM, LUSC, LUAD, SKCM, STAD, BRCA and THYM, consistent with the recognised role of ER stress signalling in the progression and adaptation of these malignancies [31, 32]. In contrast, tumours like pancreatic adenocarcinoma with reduced *EIF2AK3* expression may rely on alternative adaptive responses, such as enhanced autophagy or activation of other UPR pathways such as *ERN1* or *ATF6* [33, 34].

Furthermore, our proteomics analysis revealed that PERK protein levels are low under normal physiological conditions, in a range of human tissues, such as lymph node, pancreas and colon. These findings suggest that while *EIF2AK3* transcripts are widely expressed across human tissues, the corresponding PERK protein abundance is relatively low in many cases. This discrepancy highlights the potential influence of post-transcriptional mechanisms in modulating PERK protein levels. The translation efficiency or protein stability might be tightly regulated depending on tissue-specific demands [35, 36].

In the contexts of diseases, PERK expression was upregulated in four primary tumours (GBM, LUSC, LUAD and UCEC) but downregulated in one primary tumour (PAAD). These findings aligned with the RNA-seq data which showed significant *EIF2AK3* upregulation in GBM, LUSC and LUAD, suggesting concordant transcript-protein patterns in these tumour contexts. Such concordance has been reported for UPR components in highly stressed tumour environments [37, 38]. In contrast, the mRNA-protein discordance observed in certain tumour types, including READ, may reflect additional regulatory mechanisms operating beyond transcriptional control. Post-transcriptional processes, such as miRNA-mediated translational repression, may buffer *EIF2AK3* transcript variability and constrain PERK protein output despite elevated mRNA levels [39]. Additionally, stress-modulated ubiquitin-proteasome pathway governing regulated protein degradation, may contribute to PERK turnover dynamics [40]. These regulatory layers could enable rapid and reversible modulation of PERK signalling under fluctuating cellular stress conditions.

The study also examined the dependency of cancer cell lines on *EIF2AK3* expression. Low dependency scores observed in 17 of the 33 cell lines analysed suggest that *EIF2AK3* is not broadly essential for cancer cell survival [41], reinforcing the view that PERK dependency is not universal but context-specific. Additionally, *EIF2AK3*’s tissue-specific expression in metabolically active organs, such as the pancreas and colon, highlights the importance of carefully refining therapeutic approaches to minimise potential off-target effects [42]. Importantly, baseline dependency metrics may underestimate PERK’s functional role, as conventional 2D cultures do not replicate tumour stressors such as hypoxia and nutrient limitation, key drivers of PERK activation, particularly in stress-adapted cancers (e.g., glioblastoma, lung squamous cell carcinoma) [43, 44].

However, under stress conditions, PERK inhibition has shown antitumour activity. For example, GSK2606414 suppressed PERK-eIF2α signalling and reduced xenograft growth in lung cancer models [45], while HC-5404 enhanced responses when combined with VEGFR tyrosine kinase inhibitors in vivo [18]. Beyond proliferation, PERK has also been linked to stress-adaptive pathways, including associations with SOX2 signalling [46]. Collectively, these findings support a context-specific model in which PERK targeting is most relevant in selected, stress-adapted tumours rather than predicted by baseline *EIF2AK3* dependency alone.

The relationship between *EIF2AK3* expression and genetic alterations was also explored. Missense mutations in coding regions were associated with significant increase in *EIF2AK3* expression, and deep deletion mutations were associated with low *EIF2AK3* expression. SCNAs revealed that amplification and copy number gain events were associated with higher *EIF2AK3* expression relative to deep and shallow deletions. These results suggest the contribution of genetic factors in *EIF2AK3* regulation [47].

Patient demographic analysis indicated that while genders were not associated with *EIF2AK3* expression, ages and AJCC pathological stages were significantly associated with *EIF2AK3* expression in certain tumour types. These observations suggest that *EIF2AK3* may contribute to tumour progression in a context-dependent manner.

Pathway and GO enrichment analyses together provided a comprehensive view of the key biological processes associated with *EIF2AK3* expression across diverse tumour types. Tumours with high *EIF2AK3* expression were significantly enriched in pathways related to cell cycle, proteoglycan in cancer, and Rap1 signalling pathway, indicating increased cell motility, invasiveness, and interaction with the tumour microenvironment-features commonly linked to tumour progression and metastasis [48–50]. Proteoglycans play established roles in tumour progression by modulating ECM organisation and oncogenic signalling networks, thereby influencing processes such as invasion, angiogenesis, and therapeutic response [51, 52]. Enrichment of the Rap1 signalling pathway may similarly be relevant to cancer biology, as Rap1 activity has been linked to activation of the MAPK/ERK cascade and regulation of calcium-dependent signalling mechanisms implicated in cell migration, invasion, and metastasis [53, 54]. Other significantly enriched pathways included ubiquitin-mediated proteolysis, nucleocytoplasmic transport, collectively suggesting a tumour phenotype associated with elevated protein turnover, stress adaptation, and intracellular trafficking dynamics. Such processes are frequently linked to enhanced secretory activity, immune modulation, and adaptive stress responses observed in malignant cells [55–57]. Notably, dysregulation of the ubiquitin-proteasome system represents a well-established mechanism in cancer biology, where selective targeting of regulatory proteins, including tumour suppressors and oncoproteins, contributes to cellular homeostasis and survival [58]. Enrichment of the PI3K-Akt signalling pathway is consistent with its recognised role as a central regulator of cell growth, survival, metabolism and proliferation [59]. Furthermore, enrichment of cytoskeleton-related pathways may reflect processes involved in cell migration, invasion, and dynamic membrane protrusion formation, including structures such as lamellipodia, filopodia and invadopodia, which facilitate tumour cell motility and ECM interactions [48].

**Fig. 8:**
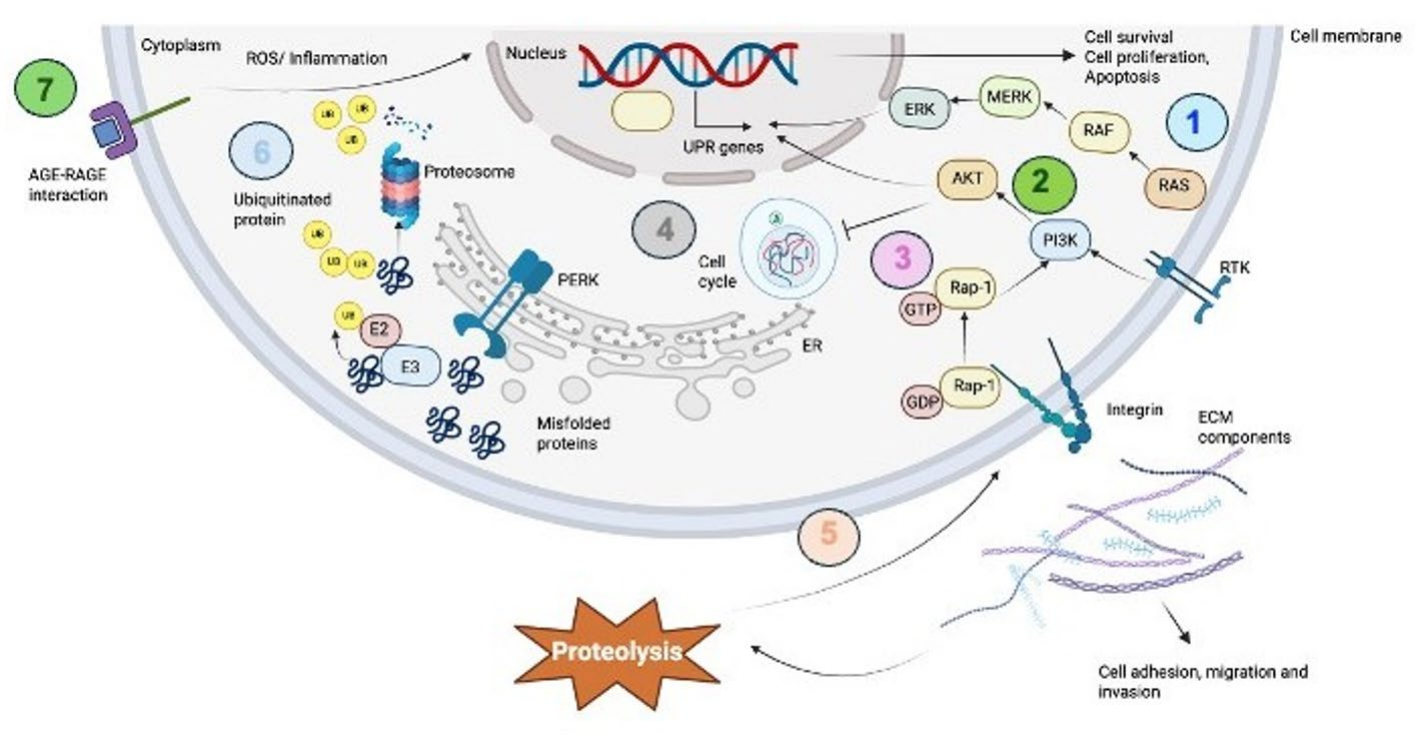
A schematic representation of the key pathways commonly enriched in the DEGs in primary tumours of high and low EIF2AK3 expression. The top enriched pathways include: (1) Ras signalling, (2) PI3K signalling, (3) Rap-1 signalling, (4) cell cycle, (5) proteoglycans in cancer and focal adhesion, (6) ubiquitin-mediated proteolysis and (7) AGE-RAGE signalling. Arrows suggest the potential interactions between pathways.

In contrast, tumours with low *EIF2AK3* expression displayed enrichment in pathways such as focal adhesion, Ras signalling, cytoskeleton regulation in muscle cells, and AGE-RAGE signalling in diabetic complications, suggesting that these tumours may depend more on adhesion-dependent survival mechanisms and oxidative stress signalling to maintain growth under less stressful conditions [60, 61]. Focal adhesion kinase often enhances cancer cell survival, motility, and invasion by suppressing anoikis, promoting stemness. It also localises to the nucleus, where it scaffolds p53 and MDM2 interactions to further support survival [62]. Whereas Ras pathway is vital for cell growth and is often hyperactive in cancers due to mutations in *RAS* genes (*KRAS*,* HRAS*,* NRAS*). These mutations trap Ras proteins in a permanently “on” state, driving uncontrolled cell division and cancer progression through pathways like RAF/MAPK [63, 64]. The enrichment of cell cycle and ubiquitin-mediated proteolysis in both high and low *EIF2AK3* groups suggests a common need for proliferation and protein quality control, though the contextual regulatory mechanisms may differ [65]. Furthermore, cytoskeleton in muscle cells was observed in both high and low *EIF2AK3* expression tumours, suggesting a shared requirement of metastasis, driven by cytoskeleton remodelling [66]. These results indicate that *EIF2AK3* expression status shapes tumour adaptive behaviour, with differential engagement of stress signalling and adhesion mechanisms (Fig. 8).

GO enrichment analysis provided additional functional context for *EIF2AK3*-associated programmes. Tumours with high *EIF2AK3* expression showed prominent enrichment of biological processes linked to adhesion and immune interaction, including positive regulation of cell adhesion, cell-cell adhesion, leukocyte adhesion, and T-cell activation. This pattern is consistent with stress-adaptive states in which PERK/UPR signalling reshapes membrane trafficking and adhesion dynamics. ER stress has been shown to impair delivery of junctional proteins (e.g., E-cadherin), promoting epithelial plasticity [67], while PERK-ATF4-CHOP signalling can constrain CD8⁺ T-cell effector function, contributing to immune dysfunction [68].

CC terms such as collagen-containing extracellular matrix, focal adhesions, and cell-substrate junctions were also enriched, suggesting active ECM remodelling and adhesion turnover, features commonly associated with invasive behaviour [69].

Enrichment of MF such as actin binding, integrin binding, and ECM structural constituents supports a mechanistic link to cytoskeletal regulation and matrix interactions, processes frequently remodelled during tumour invasion [70]. Concurrent enrichment of cytokine and growth factor binding terms is consistent with receptor-mediated signalling (e.g., TGF-β, VEGF) that shapes tumour progression and immune evasion [71]. Vesicle- and lumen-related functions further suggest elevated membrane trafficking associated with stress adaptation [72].

In contrast, tumours with low *EIF2AK3* expression were enriched for BP related to actin cytoskeletal organisation and cell-matrix interactions, including cell-substrate adhesion, actin filament organisation, and cell-matrix adhesion. These signatures indicate active coordination between cytoskeletal dynamics and ECM attachment. While disruption of adhesion pathways under persistent ER stress can promote epithelial plasticity and metastatic behaviour [73, 74], the concurrent enrichment of terms such as axonogenesis and anatomical structure homeostasis instead points towards a more structurally stabilised, less invasive phenotype. This is consistent with the central role of the ER in calcium homeostasis, a key regulator of cytoskeletal architecture and cellular stability [75].

CC enrichment, including cell-substrate junctions, actin-based projections, and actin filament bundles, aligns with PERK’s recognised functions in adhesion and cytoskeletal organisation. PERK supports cell-substrate adhesion by maintaining ER-plasma membrane contacts and F-actin architecture, whereas its loss disrupts ER-plasma membrane coupling and promotes peripheral actin accumulation [76]. Actin-based structures such as filopodia and lamellipodia are closely linked to ER stress, with disturbances in ER homeostasis (e.g., protein misfolding or Ca²⁺ imbalance) driving actin remodelling and morphological change [77]. Enrichment of basal cell and basal membrane terms further highlights processes at cell-matrix interfaces, where integrin-mediated adhesions connect the actin cytoskeleton to the ECM and regulate polarity, migration, and survival signalling [78].

MF enrichment, including glycosaminoglycan (GAG) binding, actin binding, and ECM structural constituents, points to coordinated regulation of matrix signalling and cytoskeletal integrity. Sulfated GAGs are known to modulate extracellular pathways, while integrin-actin coupling transduces mechanical cues that stabilise adhesion and ECM architecture [79, 80]. Together, these features suggest a tumour state prioritising adhesion integrity and matrix interaction rather than stress-driven plasticity, consistent with a homeostatic role for *EIF2AK3* under lower-stress conditions [81]. Additional enrichment of growth factor and integrin binding functions further supports adhesion-mediated signalling networks that reinforce cytoskeletal and ECM integrity [82, 83]. These findings indicate that *EIF2AK3* may act as a context-dependent regulator, driving either tumour aggressiveness or maintaining cellular homeostasis, depending on its expression level and the cellular environment.

Survival analysis demonstrated tumour-specific effects of *EIF2AK3* expression. Across the pan-cancer cohort, high *EIF2AK3* levels were associated with improved overall and disease-free survival (HR < 1), suggesting a broadly protective trend. However, in BRCA and LGG, high *EIF2AK3* expression correlated with poorer overall survival (HR > 1), indicating a context-dependent role in tumour biology. In BRCA, elevated *EIF2AK3* (PERK) expression has been linked to adverse clinicopathological features and unfavourable prognosis, consistent with a pro-tumorigenic contribution of UPR signalling and tumour stress adaptation. PERK activity has also been implicated in shaping the immune microenvironment, including effects on myeloid and T-cell function [84, 85]. PTEN loss, a common event in breast cancer that enhances PI3K/AKT signalling, may further interact with stress-response pathways relevant to PERK-mediated effects [86]. In LGG, ER stress and UPR activation are similarly associated with progression and outcome [84]. Mechanistic studies show that PERK can regulate metabolic programmes; for example, in IDH-mutant LGG models, PERK modulates cholesterol homeostasis via LDLR, ABCA1, and APOE, accompanied by changes in microglial/macrophage phenotypes and reduced invasiveness [87].

Furthermore, hierarchical clustering revealed an *EIF2AK3*-associated gene cluster commonly upregulated in many cancers. Functional enrichment and PPI network analyses showed that this cluster was strongly associated with pathways involved in cell adhesion, epithelial development and cytoskeletal remodelling, hallmarks of invasive tumour behaviour [88, 89].

Notably, GBM and LGG exhibited a distinct expression pattern characterised by downregulation of the *EIF2AK3*-associated gene cluster. This suggests that *EIF2AK3*-related pathways may function differently in GBM and LGG. Despite *EIF2AK3* upregulation relative to normal tissue, the coordinated PERK-associated transcriptional programme displayed comparatively lower expression, consistent with tumour-specific regulatory or stress-adaptation mechanisms.

Beyond cancer-related processes, the *EIF2AK3*-related cluster was also enriched in genes linked to neurodegenerative and genetic disease pathways, suggesting broader associations between PERK signalling and cellular stress response networks. Enrichment of pathways related to tight junction organisation and ECM regulation further aligns with established roles of stress-adaptive signalling in tissue architecture and tumour-associated remodelling processes [90, 91].

## Limitations

While this study provides a comprehensive multi-omics analysis of *EIF2AK3* across diverse tumour types, several limitations should be acknowledged. First, reliance on publicly available transcriptomic and proteomic datasets may introduce variability due to sample heterogeneity, cohort composition, and technical differences across platforms. Although cross-dataset harmonisation strategies were applied where appropriate, residual confounding cannot be fully excluded. Second, the present findings are correlative and therefore do not establish causal or mechanistic relationships between *EIF2AK3* expression and tumour biology.

To strengthen biological inference, experimental validation will be essential. In particular, functional studies using *EIF2AK3* knockdown or overexpression in cancer cell lines, CRISPR-mediated gene editing, and pharmacological inhibition can help delineate its mechanistic role in regulating ER stress, apoptosis and tumour progression. Moreover, in vivo models will be essential for evaluating systemic effects, tumour microenvironment interactions, and the broader physiological consequences of *EIF2AK3* (PERK) modulation.

Finally, the interpretation of multi-omics associations in cancer increasingly benefits from integrative computational frameworks, including machine learning and cross-disease network analyses. Recent studies have demonstrated how multi-omics integration can refine prognostic modelling, identify shared molecular mechanisms, and prioritise candidate targets across cancer types and complex diseases [92–95]. Incorporating such approaches in future investigations may further enhance the robustness and translational relevance of *EIF2AK3* (or PERK)-focused analyses.

## Conclusion

This study highlights the context-dependent expression patterns and clinical associations of *EIF2AK3* (PERK) across human cancers. High *EIF2AK3* expression is associated with poorer survival in selected tumour types, particularly BRCA and LGG, and corresponds with enrichment of pathways linked to ER stress and cell adhesion. Conversely, lower *EIF2AK3* expression is associated with enrichment of adhesion-, cytoskeleton-, and microenvironment-related pathways. While the present analyses are correlative and do not establish mechanistic causality, the findings indicate that PERK-associated signalling is associated with distinct stress- and adhesion-related transcriptional programmes in specific biological contexts. Collectively, these observations underscore the need for further functional investigation to clarify whether PERK represents a context-specific biological dependency or regulatory node, rather than a uniform feature across tumour types, particularly in malignancies characterised by elevated ER stress, thereby supporting further evaluation of PERK as a candidate therapeutic target in cancer.

## Supplementary Information

Below is the link to the electronic supplementary material.

Supplementary Material 1.

Supplementary Material 2.

## Data Availability

All datasets used in this study are publicly available through the respective portals as described in the Methods section. RNA-seq data for primary tumours and matched controls were obtained from the UCSC Xena Functional Genomics Browser using TCGA and TARGET datasets. GTEx datasets were downloaded from OncoDB. Normalised demographic and clinical data were accessed through the TCGA PanCancer cohort via cBioPortal. Protein abundance and cell lines and tissue abundance were analysed via ProteomicsDB, PaxDb, and protein MS expression from CPTAC were downloaded from Human Protein Atlas. Dependency scores for tumour cell lines were retrieved from the Cancer Dependency Map (DepMap). Mutation and copy number alteration data were obtained from cBioPortal. Survival analysis was performed via GEPIA2 platform. Protein-protein interaction network was performed using the STRING database. Functional enrichment analyses were conducted using the Diseases, Gene Ontology (GO) and Reactome pathway databases. The links for the datasets and resources used in this study are included in supplementary information files. The code used in the analysis will be available from the corresponding author upon reasonable request.

## Abbreviations

ACC: Adrenocortical carcinoma
AJCC: American joint committee on cancer
AML: Acute myeloid leukaemia
ANCOVA: Analysis of covariance
ANOVA: Analysis of variance
ATF6α: Activating transcription factor 6 alpha
BH: Benjamini-hochberg
BLCA: Bladder urothelial carcinoma
BP: Biological processes
BRCA: Breast invasive carcinoma
CC: Cellular components
CESC: Cervical squamous cell carcinoma and endocervical adenocarcinoma
CHOL: Cholangiocarcinoma
COAD: Colon adenocarcinoma
CPTAC: Clinical proteomic tumour analysis consortium
CRs: Coding regions
CRC: Colorectal cancer
DFS: Disease-free survival
DEGs: Differentially expressed genes
DepMap: Dependency map
DLBC: Diffuse large B-cell lymphoma
ECM: Extracellular matrix
EIF2AK3: Eukaryotic translation initiation factor 2 alpha kinase 3 (gene encoding PERK)
ER: Endoplasmic reticulum
ERAD: ER-associated degradation
ESCA: Oesophageal carcinoma
FDR: False discovery rate
GAGs: Glycosaminoglycans
GBM: Glioblastoma multiforme
GDC: Genomic data commons
GEPIA2: Gene expression profiling interactive analysis 2
GO: Gene ontology
GTEx: Genotype-tissue expression
HEK: Human embryonic kidney
HNSC: Head and neck squamous cell carcinoma
HR: Hazard ratio
IQR: Interquartile range
IRE1α: Inositol-requiring enzyme 1 alpha
KEGG: Kyoto encyclopedia of genes and genomes
KICH: Kidney chromophobe
KIRC: Kidney renal clear cell carcinoma
KIRP: Kidney renal papillary cell carcinoma
LC-MS/MS: Liquid chromatography-tandem mass spectrometry
LGG: Lower grade glioma
LIHC: Liver hepatocellular carcinoma
Log_2_ FC: Log_2_ fold change
LUSC: Lung squamous cell carcinoma
LUAD: Lung adenocarcinoma
MF: Molecular functions
MMPs: Matrix metalloproteinases
NATs: Normal adjacent tissues
nRPX: Normalised relative protein expression
NCRs: Non-coding regions
OS: Overall survival
OV: Ovarian serous cystadenocarcinoma
OncoDB: Oncogenomic database
PAAD: Pancreatic adenocarcinoma
PCPG: Pheochromocytoma and paraganglioma
PERK: Protein kinase R-like endoplasmic reticulum kinase
PPI: Protein-protein interaction
PRAD: Prostate adenocarcinoma
RSEM: RNA-seq by expectation maximisation
READ: Rectal adenocarcinoma
SCNA: Somatic copy number alteration
SKCM: Skin cutaneous melanoma
STAD: Stomach adenocarcinoma
STRING: Search tool for the retrieval of interacting genes/proteins
TARGET: Therapeutically applicable research to generate effective treatments
TCGA: The cancer genome atlas
TGCT: Testicular germ cell tumour
THCA: Thyroid carcinoma
THYM: Thymoma
TMT: Tandem mass tags
UCEC: Uterine corpus endometrioid carcinoma
UPR: Unfolded protein response
