# Supplementary material for: Pan-cancer analysis reveals differential PERK expression across tumour types and its potential as a therapeutic target
Source: Discov Oncol. 2026 Mar 25;17:681. doi: 10.1007/s12672-026-04910-8 (PMC13136445; doi:10.1007/s12672-026-04910-8)
Supplement: Supplementary file 1 — Supplementary Material 1. [file 12672_2026_4910_MOESM1_ESM.pdf]

Supplementary Figures

Pan-cancer bioinformatic analysis identifies PERK as a potential therapeutic target

Shivam Kumar[1], Namshik Han[2,3], Georgia Tsagkogeorga[3,4], Murphy Lam Yim Wan[1,5*]

[1]School of Medicine, Pharmacy and Biomedical Sciences, Faculty of Science and Health, University of Portsmouth, Portsmouth, PO1 2DT, United Kingdom

[2]Cambridge Centre for AI in Medicine, University of Cambridge, Cambridge CB2 0QQ, United Kingdom

[3]Milner Therapeutics Institute, University of Cambridge, Cambridge CB4 0WS, United Kingdom

[4]STORM Therapeutics Ltd., Babraham Research Campus, Cambridge CB22 3AT, United Kingdom

[5]Department of Laboratory Medicine, Division of Microbiology, Immunology and Glycobiology, Lund University, 221 84 Lund, Sweden

Address for correspondence:
Dr Murphy Lam Yim Wan
Department of Laboratory Medicine, Division of Microbiology, Immunology and Glycobiology, Lund University, 221 84 Lund, Sweden
School of Medicine, Pharmacy and Biomedical Sciences, Faculty of Science and Health, University of Portsmouth, Portsmouth, PO1 2DT, United Kingdom
Email: murphy.wan@med.lu.se

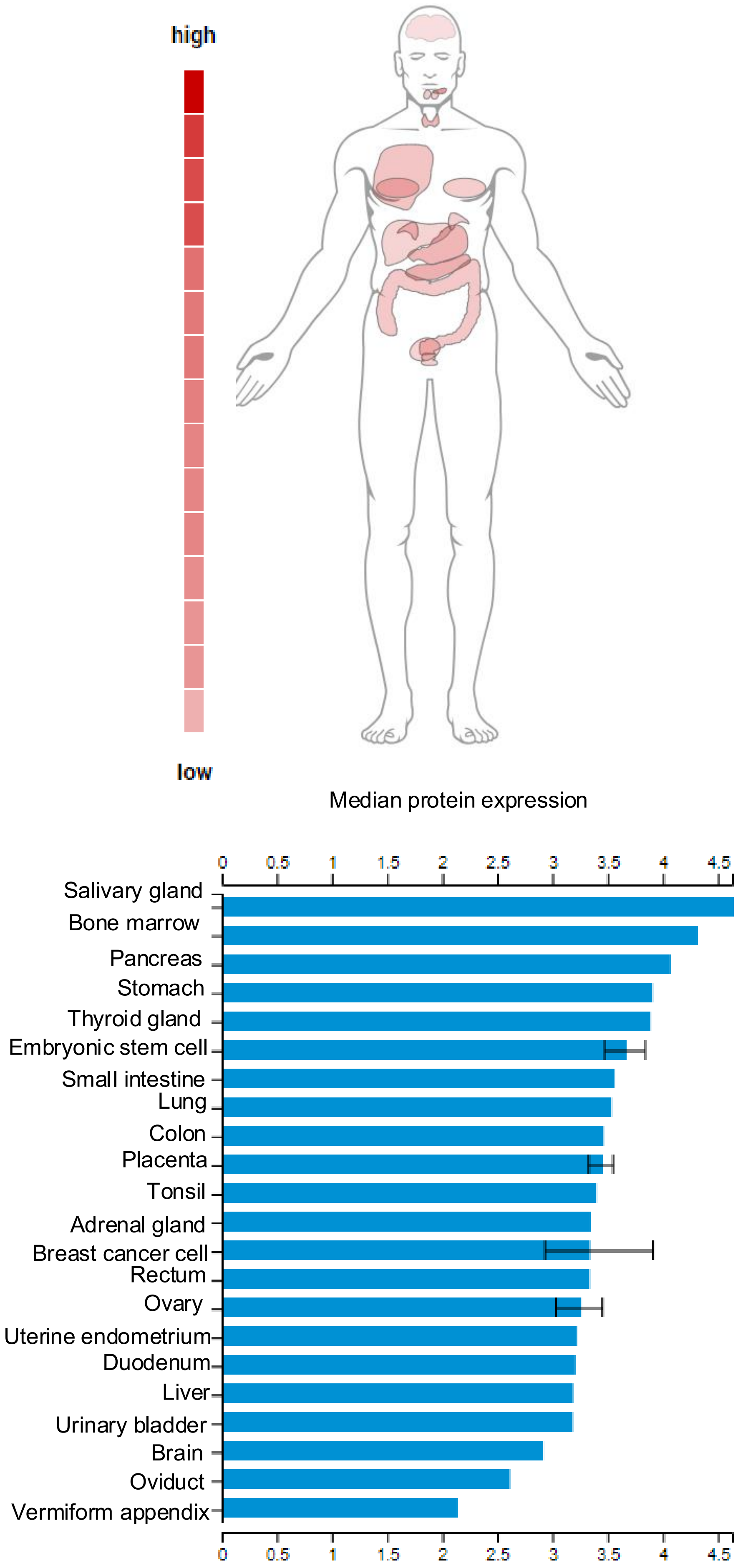

Supplementary Fig. S1. Full-length PERK protein expression in normal tissues. Data were obtained from Proteomics DB.

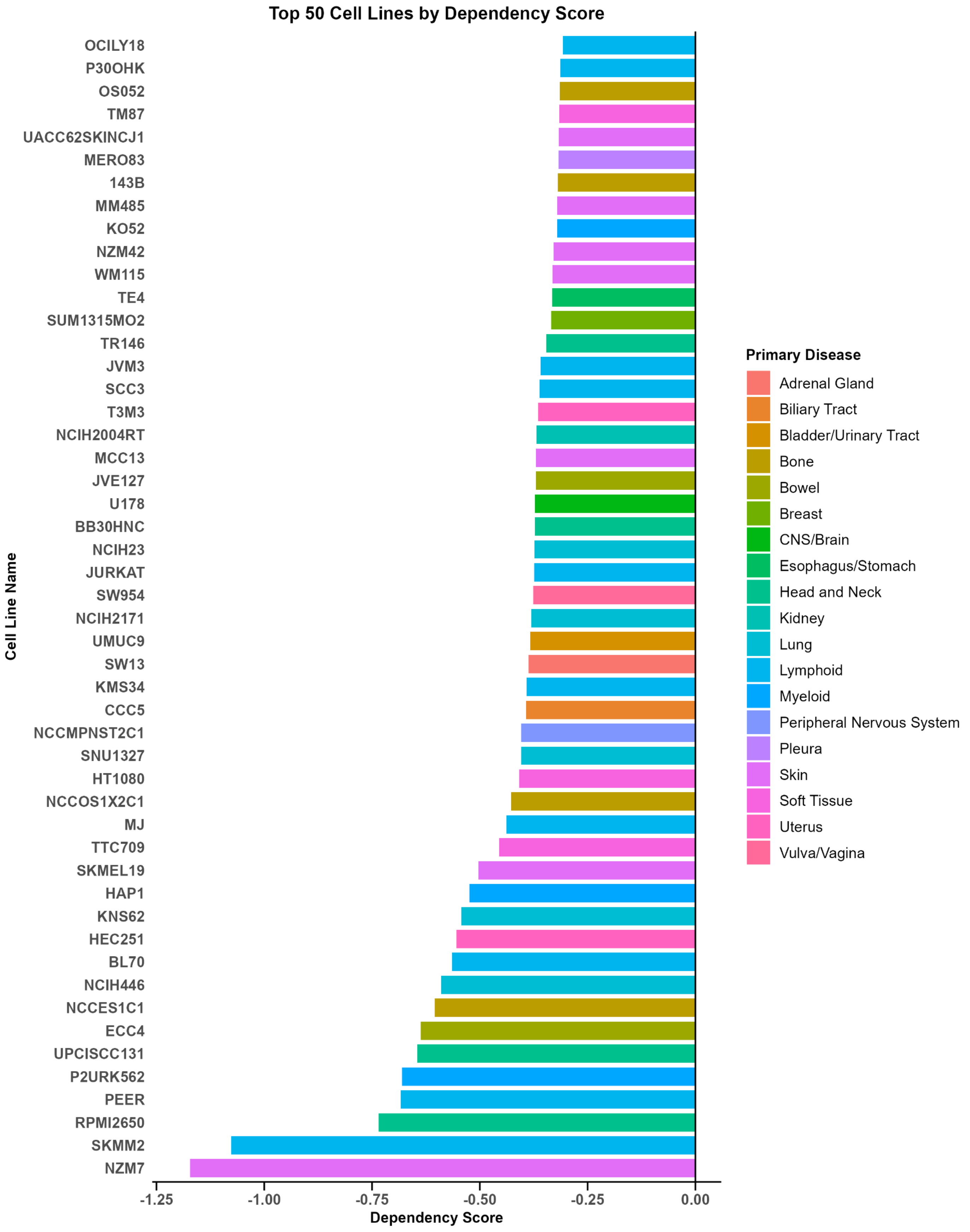

Supplementary Fig. S2. Dependency scores for *EIF2AK3* across cancer cell lines using data from the Dependency Map (DepMap). Gene effect scores, normalised using the DEMETER2 or CERES algorithms, are scaled for comparability across genes: a score of 0 corresponds to the median of non-essential genes, while a score of −1 corresponds to the median of essential genes. The top 50 cell lines ranked by *EIF2AK3* dependency are shown, coloured by primary disease type. Boxplots display the median dependency scores ± interquartile range (IQR) across cancer types, with whiskers indicating variability among cell lines. No statistical significance of dependency is found within the dataset.

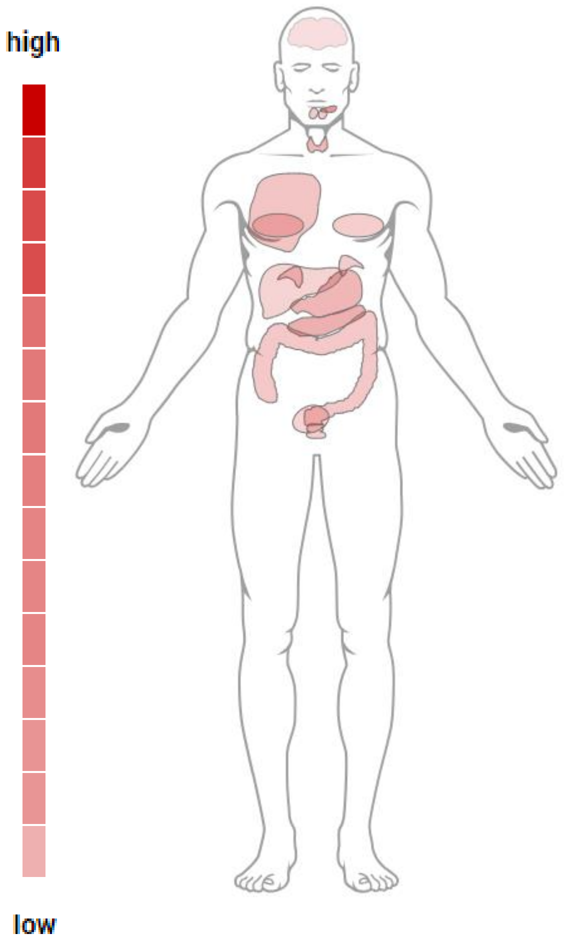

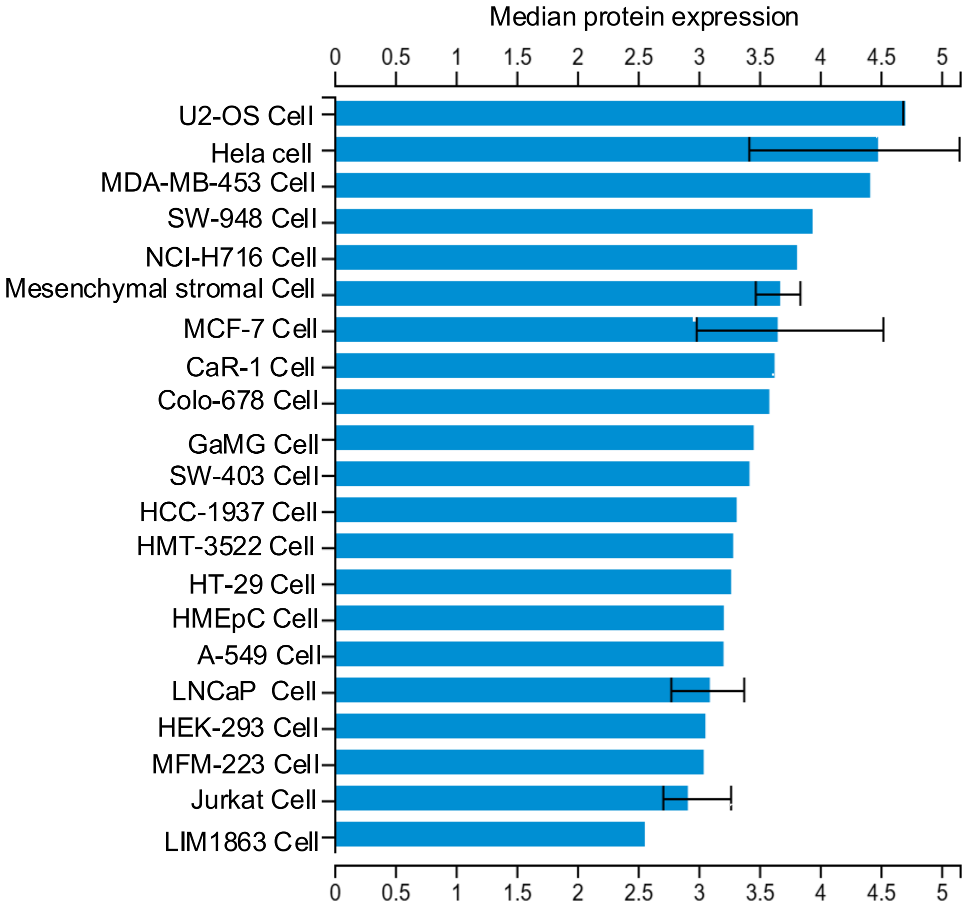

Supplementary Fig. S3. PERK protein expression in cancer cell lines. Data were obtained from Proteomics DB.

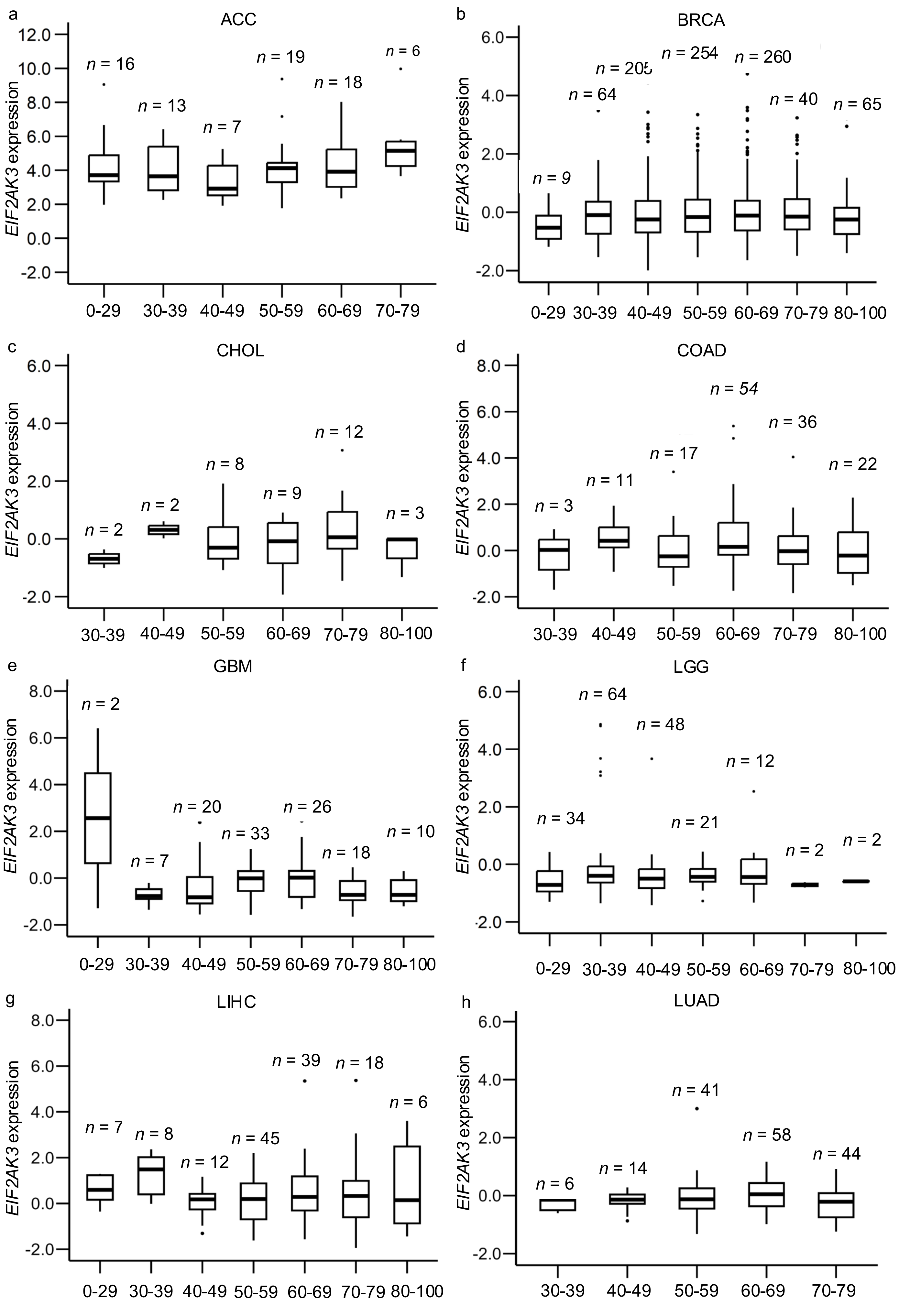
a
ACC
EIF2AK3 expression
n = 16
n = 13
n = 7
n = 19
n = 18
n = 6
0-29
30-39
40-49
50-59
60-69
70-79
b
BRCA
EIF2AK3 expression
n = 9
n = 64
n = 254
n = 260
n = 40
n = 65
0-29
30-39
40-49
50-59
60-69
70-79
80-100
c
CHOL
EIF2AK3 expression
n = 2
n = 2
n = 8
n = 9
n = 12
n = 3
30-39
40-49
50-59
60-69
70-79
80-100
d
COAD
EIF2AK3 expression
n = 3
n = 11
n = 17
n = 54
n = 36
n = 22
30-39
40-49
50-59
60-69
70-79
80-100
e
GBM
EIF2AK3 expression
n = 2
n = 7
n = 20
n = 33
n = 26
n = 18
n = 10
0-29
30-39
40-49
50-59
60-69
70-79
80-100
f
LGG
EIF2AK3 expression
n = 34
n = 64
n = 48
n = 21
n = 12
n = 2
n = 2
0-29
30-39
40-49
50-59
60-69
70-79
80-100
g
LIHC
EIF2AK3 expression
n = 7
n = 8
n = 12
n = 45
n = 39
n = 18
n = 6
0-29
30-39
40-49
50-59
60-69
70-79
80-100
h
LUAD
EIF2AK3 expression
n = 6
n = 14
n = 41
n = 58
n = 44
30-39
40-49
50-59
60-69
70-79

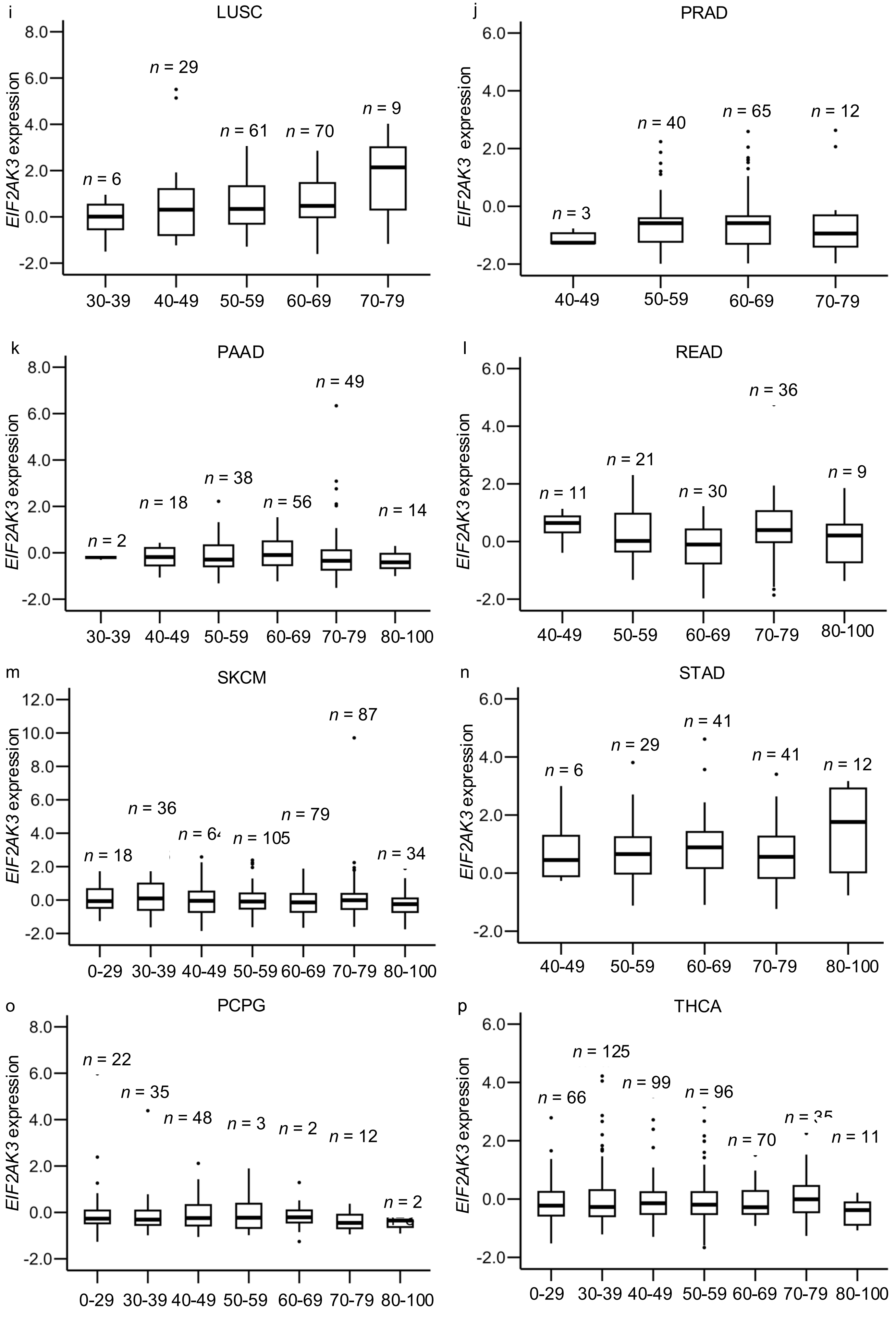

i
LUSC
EIF2AK3 expression
n = 6
n = 29
n = 61
n = 70
n = 9
30-39
40-49
50-59
60-69
70-79
j
PRAD
EIF2AK3 expression
n = 3
n = 40
n = 65
n = 12
40-49
50-59
60-69
70-79
k
PAAD
EIF2AK3 expression
n = 2
n = 18
n = 38
n = 56
n = 49
n = 14
30-39
40-49
50-59
60-69
70-79
80-100
l
READ
EIF2AK3 expression
n = 11
n = 21
n = 30
n = 36
n = 9
40-49
50-59
60-69
70-79
80-100
m
SKCM
EIF2AK3 expression
n = 18
n = 36
n = 64
n = 105
n = 79
n = 87
n = 34
0-29
30-39
40-49
50-59
60-69
70-79
80-100
n
STAD
EIF2AK3 expression
n = 6
n = 29
n = 41
n = 41
n = 12
40-49
50-59
60-69
70-79
80-100
o
PCPG
EIF2AK3 expression
n = 22
n = 35
n = 48
n = 3
n = 2
n = 12
n = 2
0-29
30-39
40-49
50-59
60-69
70-79
80-100
p
THCA
EIF2AK3 expression
n = 66
n = 125
n = 99
n = 96
n = 70
n = 35
n = 11
0-29
30-39
40-49
50-59
60-69
70-79
80-100

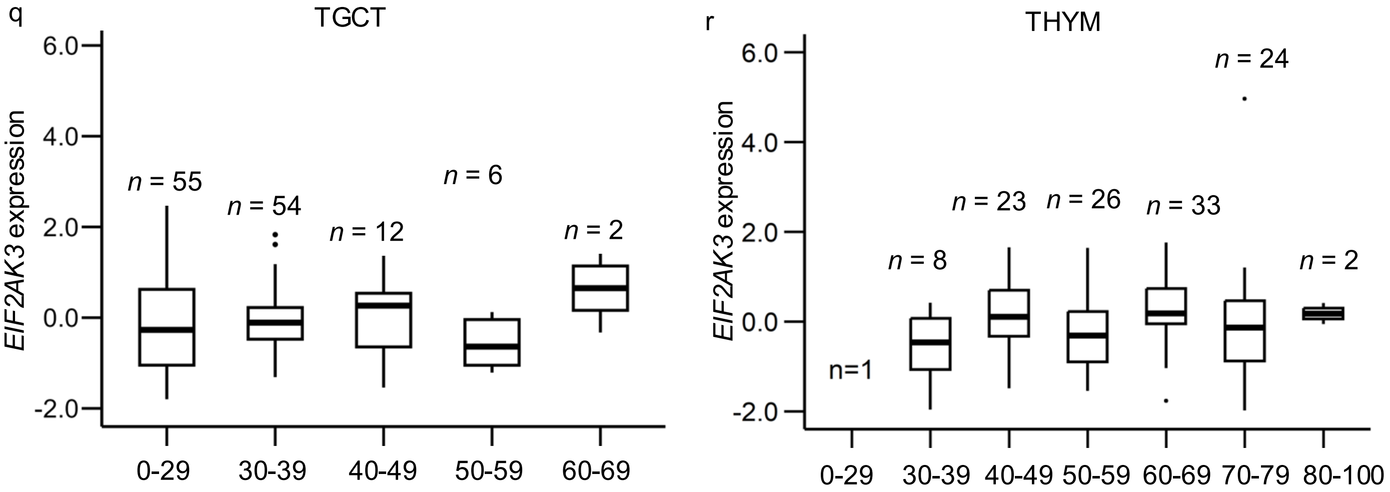

Supplementary Fig. S4. Comparison of *EIF2AK3* expression across age groups within primary tumour cohorts. RNA-seq data were Log_2 (RSEM+1)-transformed, and comparisons were performed within the following tumour cohorts: (a) ACC, (b) BRCA, (c) CHOL, (d) COAD, (e) GBM, (f) LGG, (g) LIHC, (h) LUAD (i) LUSC, (j) PRAD, (k) PAAD, (l) READ, (m) SKCM, (n) STAD, (o) PCPG, (p) THCA, (q) TGCT and (r) THYM. Statistical analysis was conducted using the Kruskal-Wallis test followed by Dunn's multiple comparisons test. Data are presented as mean ± SEM, with sample sizes (*n*) indicated on the plots.

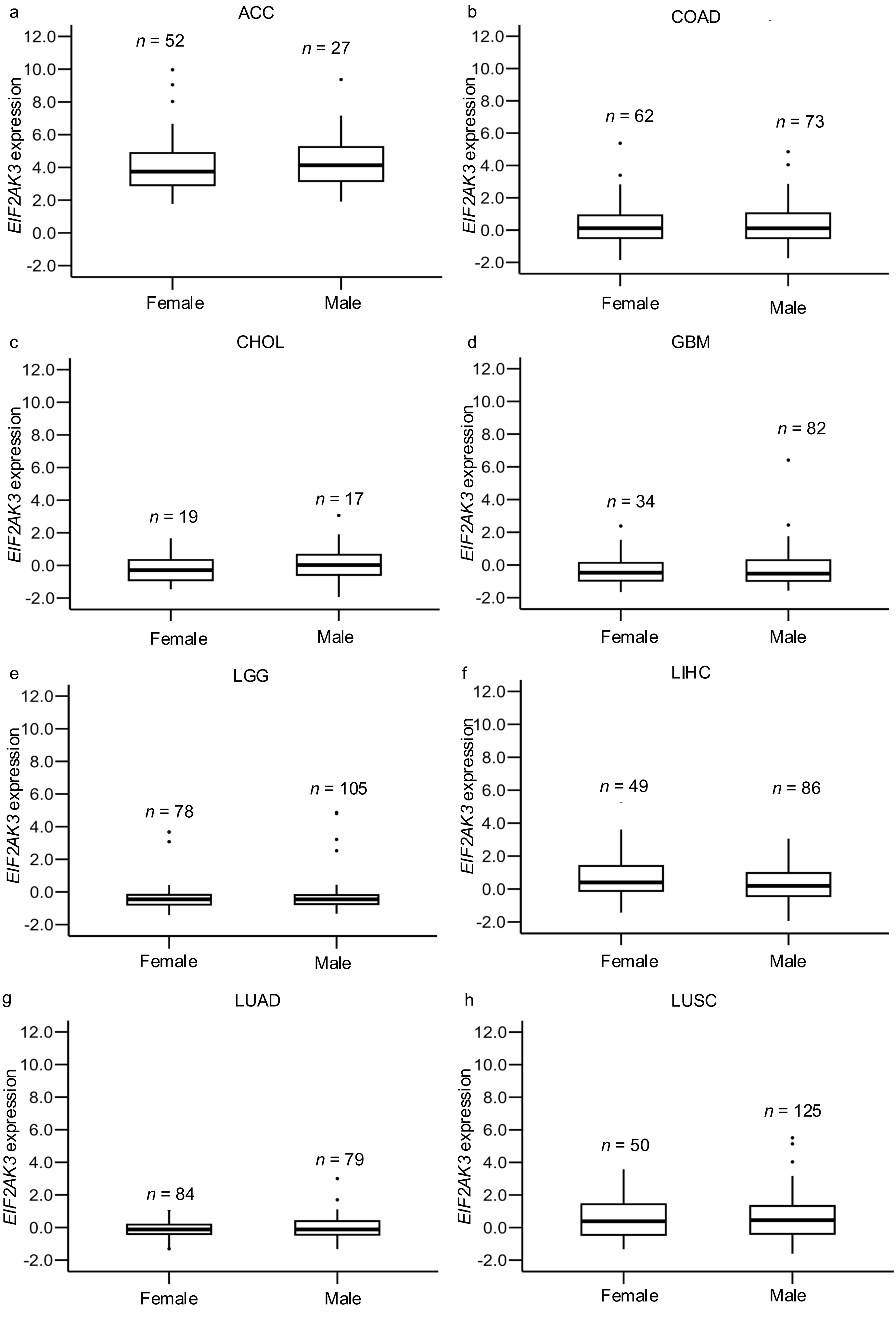
a
ACC
n = 52
n = 27
b
COAD
n = 62
n = 73
c
CHOL
n = 19
n = 17
d
GBM
n = 34
n = 82
e
LGG
n = 78
n = 105
f
LIHC
n = 49
n = 86
g
LUAD
n = 84
n = 79
h
LUSC
n = 50
n = 125
EIF2AK3 expression
Female
Male
12.0
10.0
8.0
6.0
4.0
2.0
0.0
-2.0

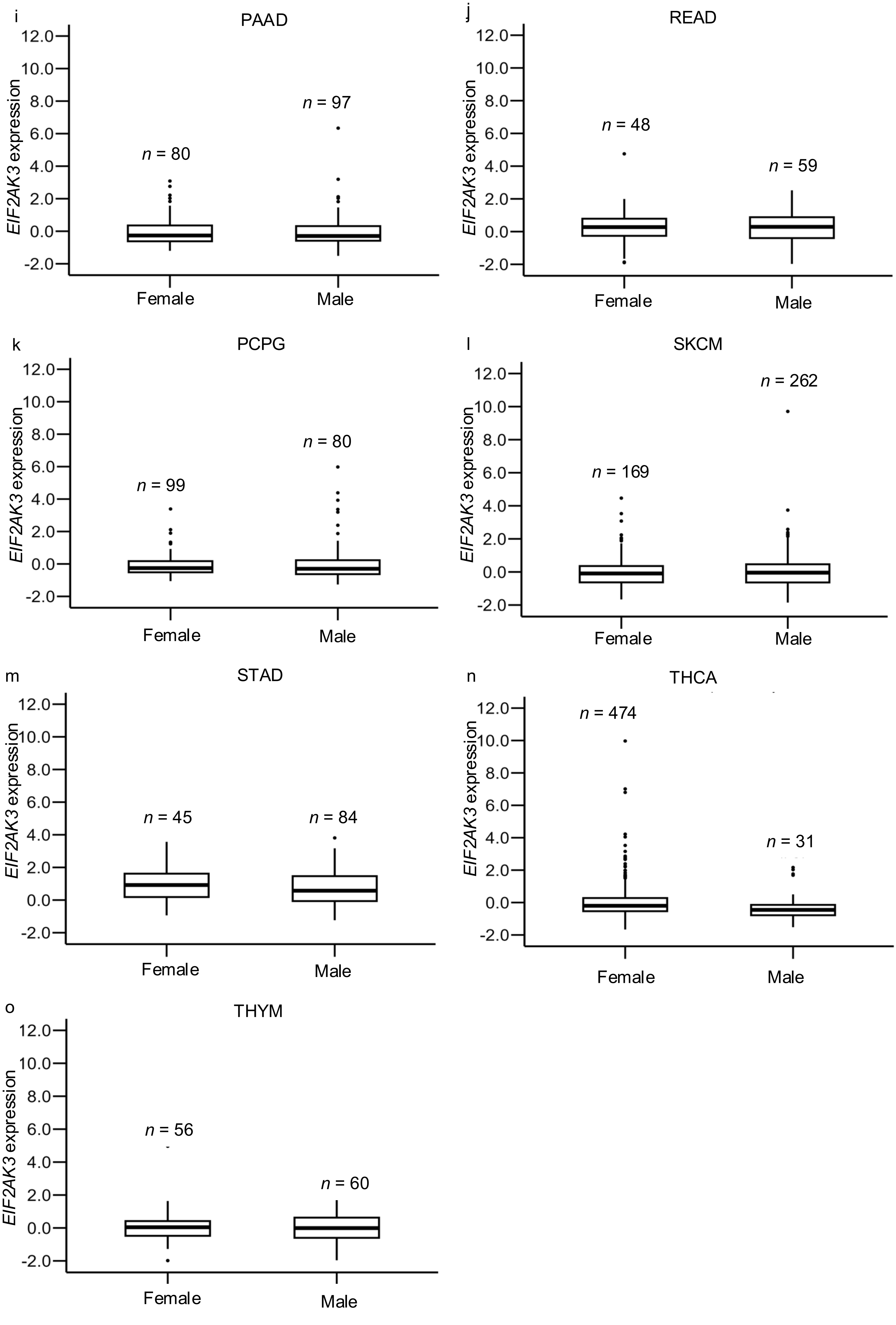
i
PAAD
n = 80
n = 97
Female
Male
j
READ
n = 48
n = 59
Female
Male
k
PCPG
n = 99
n = 80
Female
Male
l
SKCM
n = 169
n = 262
Female
Male
m
STAD
n = 45
n = 84
Female
Male
n
THCA
n = 474
n = 31
Female
Male
o
THYM
n = 56
n = 60
Female
Male
EIF2AK3 expression
12.0
10.0
8.0
6.0
4.0
2.0
0.0
-2.0

Supplementary Fig. S5. Comparison of *EIF2AK3* expression across gender groups within primary tumour cohorts. RNA-seq data were Log_2 (RSEM+1)-transformed, and comparisons were performed within the following tumour cohorts: (a) ACC, (b) COAD, (c) CHOL, (d) GBM, (e) LGG, (f) LIHC, (g) LUAD, (h) LIHC, (i) PAAD, (j) READ, (k) PCPG, (l) SKCM, (m) STAD, (n) THCA and (o) THYM. Statistical analysis was conducted using the Welch's t-test. Data are presented as mean ± SEM, with sample sizes (*n*) indicated on the plots.

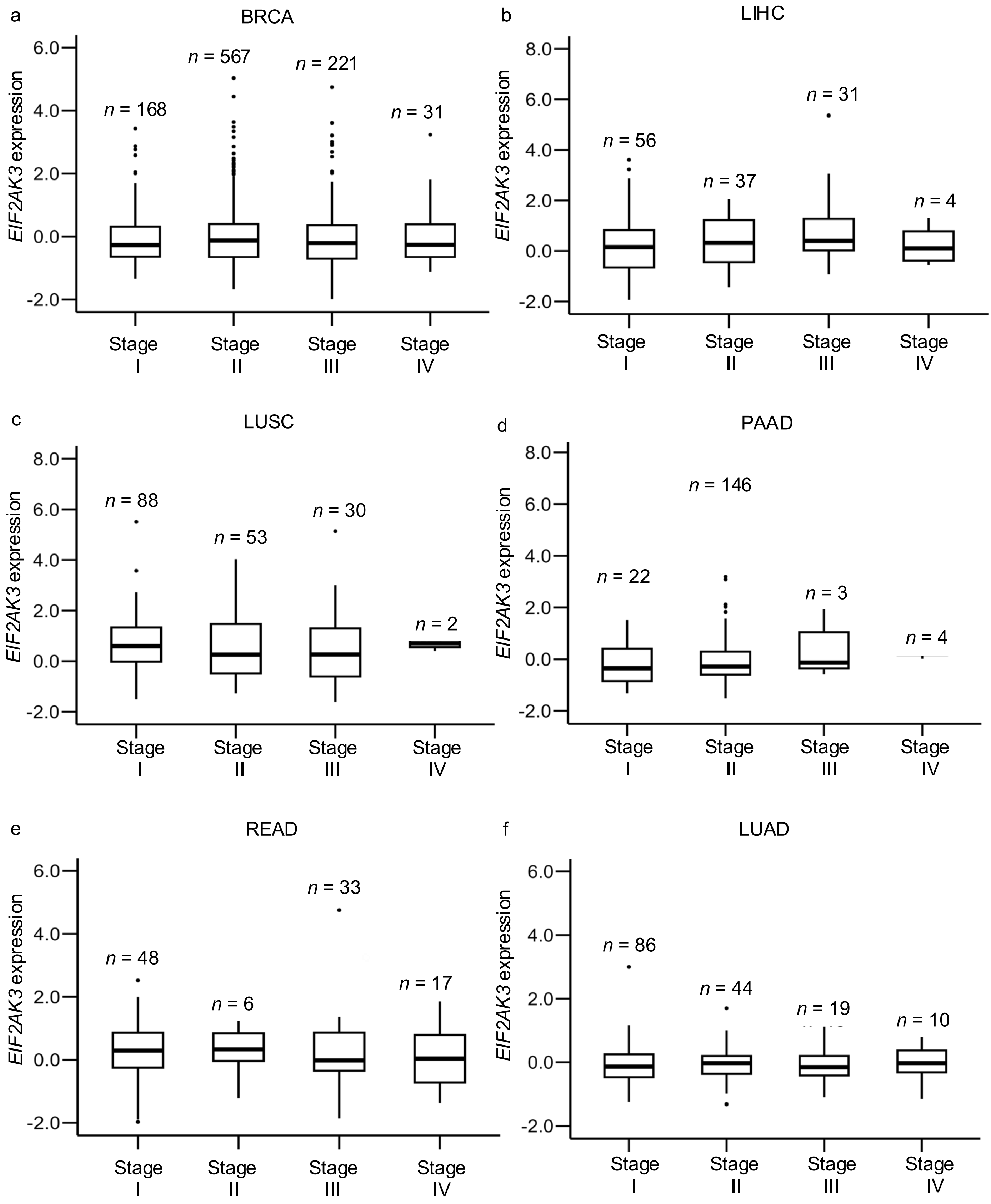

a
BRCA
n = 168
n = 567
n = 221
n = 31
b
LIHC
n = 56
n = 37
n = 31
n = 4
c
LUSC
n = 88
n = 53
n = 30
n = 2
d
PAAD
n = 22
n = 146
n = 3
n = 4
e
READ
n = 48
n = 6
n = 33
n = 17
f
LUAD
n = 86
n = 44
n = 19
n = 10
EIF2AK3 expression
Stage I
Stage II
Stage III
Stage IV

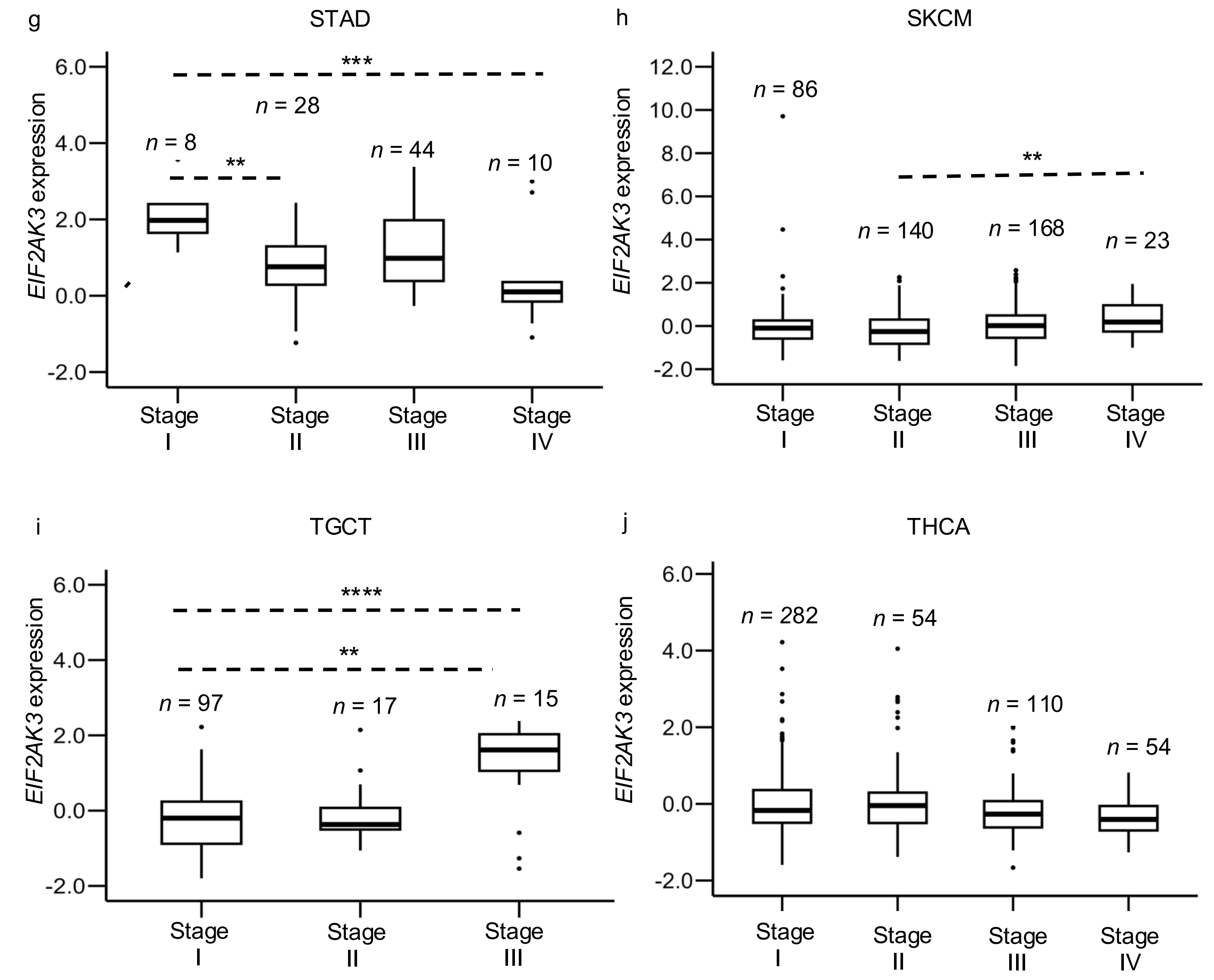

Supplementary Fig. S6. Comparison of *EIF2AK3* expression across pathological Stages (Stage I to Stage IV) groups within primary tumour cohorts. RNA-seq data were Log_2 (RSEM+1)-transformed, and comparisons were performed for (a) BRCA, (b) LIHC, (c) LUSC, (d) PAAD, (e) READ, (f) LUAD, (g) STAD, (h) SKCM, (i) TGCT and (j) THCA. Statistical analysis was conducted using the Kruskal-Wallis with Dunn's comparisons. Data are presented as mean ± SEM, with sample sizes (*n*) indicated on the plots. ** P <0.01, *** P <0.001 and ****P<0.0001

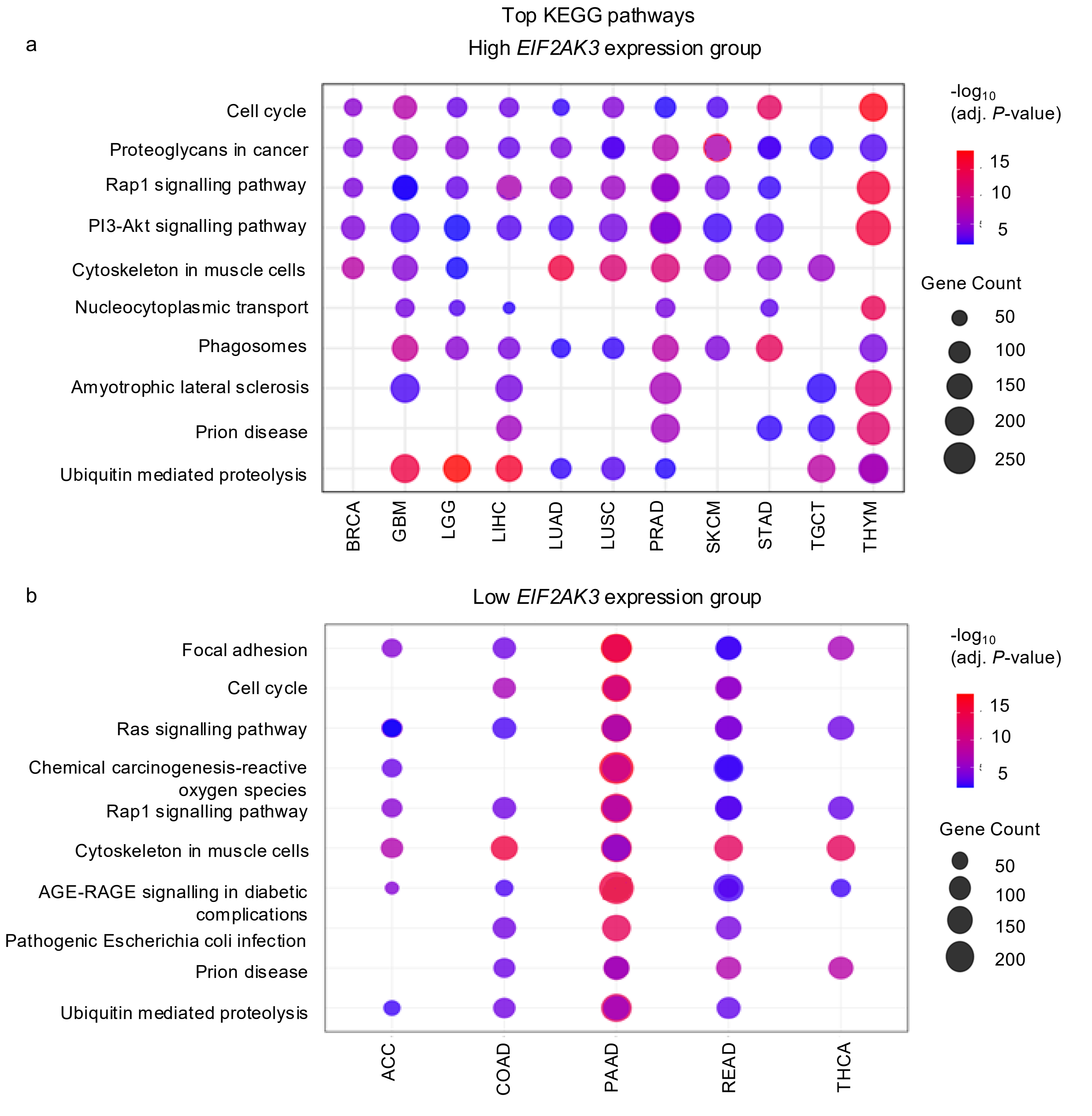

Supplementary Fig. S7. Top KEGG pathway enriched in differentially expressed genes (DEGs) in primary tumours with (a) high and (b) low *EIF2AK3* expression levels. Each bubble represents a pathway enriched in a specific cancer type. Data are represented as ($-\log_{10}$[adj. *P*-value]). Bubble size indicates the number of genes associated with each term, while bubble colour reflects the magnitude of the combined enrichment score.

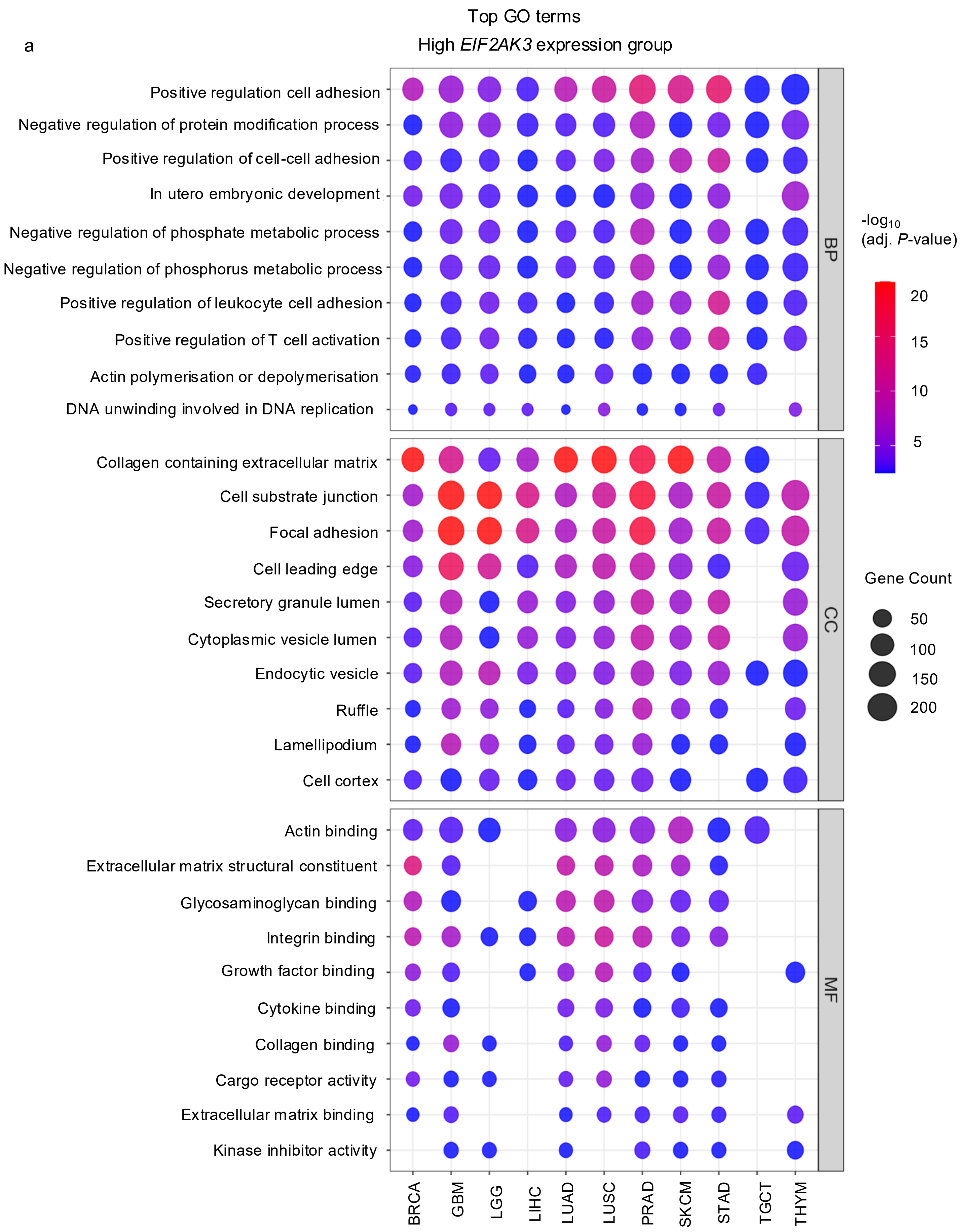
a
Top GO terms
High EIF2AK3 expression group
Positive regulation cell adhesion
Negative regulation of protein modification process
Positive regulation of cell-cell adhesion
In utero embryonic development
Negative regulation of phosphate metabolic process
Negative regulation of phosphorus metabolic process
Positive regulation of leukocyte cell adhesion
Positive regulation of T cell activation
Actin polymerisation or depolymerisation
DNA unwinding involved in DNA replication
Collagen containing extracellular matrix
Cell substrate junction
Focal adhesion
Cell leading edge
Secretory granule lumen
Cytoplasmic vesicle lumen
Endocytic vesicle
Ruffle
Lamellipodium
Cell cortex
Actin binding
Extracellular matrix structural constituent
Glycosaminoglycan binding
Integrin binding
Growth factor binding
Cytokine binding
Collagen binding
Cargo receptor activity
Extracellular matrix binding
Kinase inhibitor activity
BP
CC
MF
BRCA
GBM
LGG
LIHC
LUAD
LUSC
PRAD
SKCM
STAD
TGCT
THYM
-log10
(adj. P-value)
20
15
10
5
Gene Count
50
100
150
200

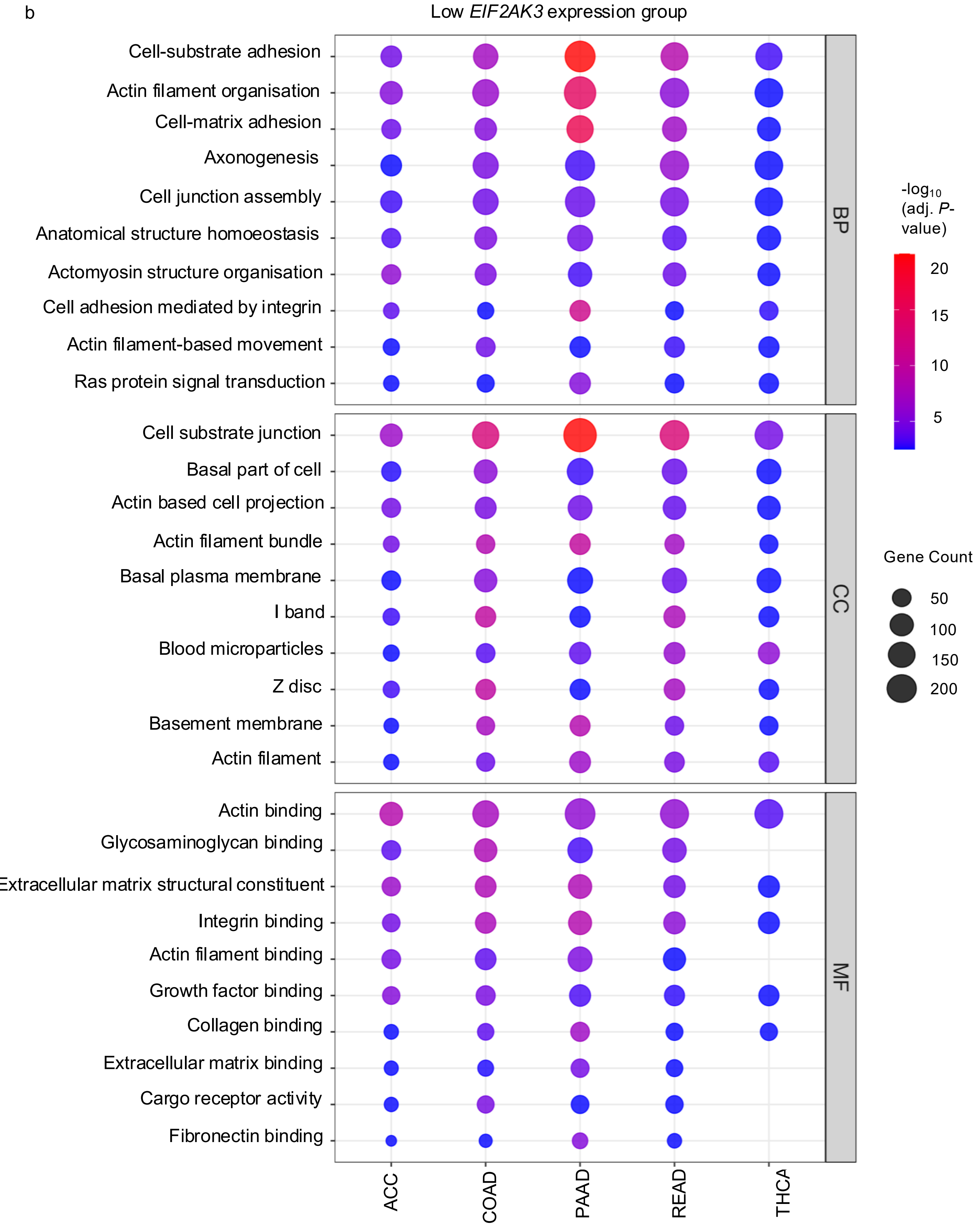

Supplementary Fig. S8. GO terms enriched in differentially expressed genes (DEGs) in primary tumours with (a) high and (b) low *EIF2AK3* expression. BP: Biological process; CC: cellular component; MF: molecular function. Each bubble represents a GO term enriched in a specific cancer type. Data are represented as $-\log_{10}$(adj. *P*-value). Bubble size indicates the number of genes associated with each term, while bubble colour reflects the magnitude of $-\log_{10}$(adj. *P*-value).

Overall survival Disease-free survival

ACC

a

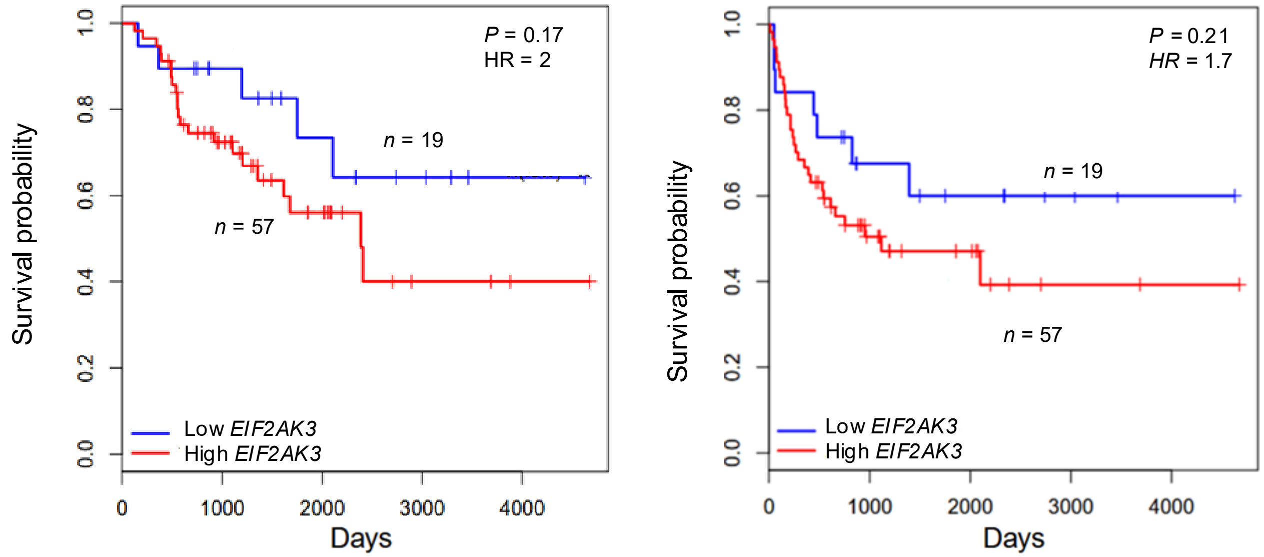

COAD

b

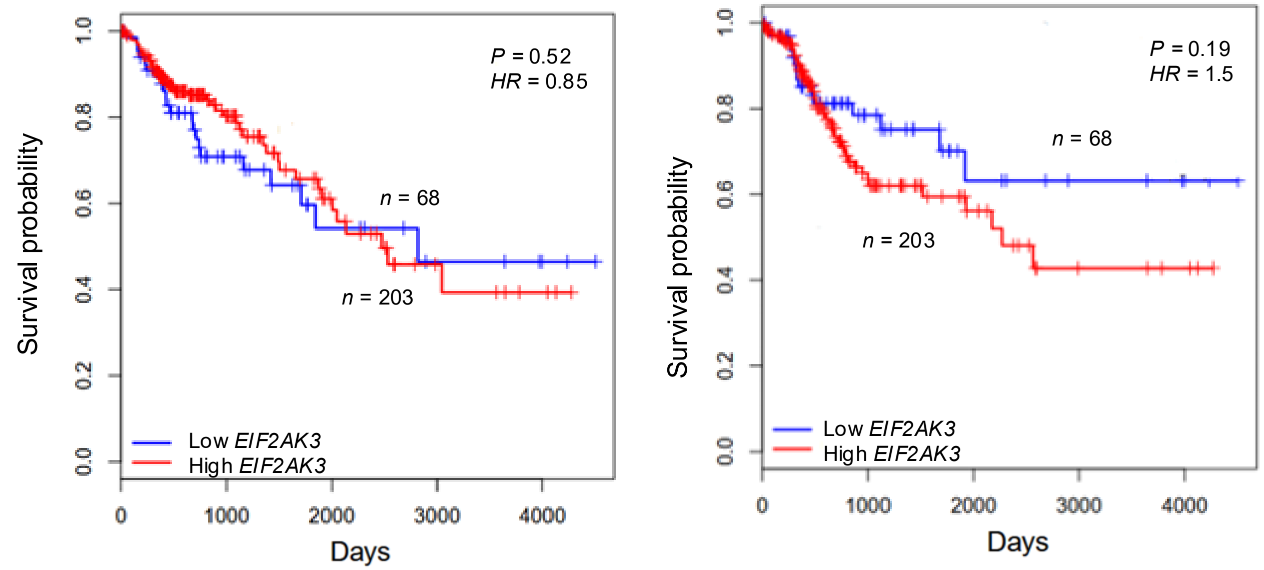

CHOL

c

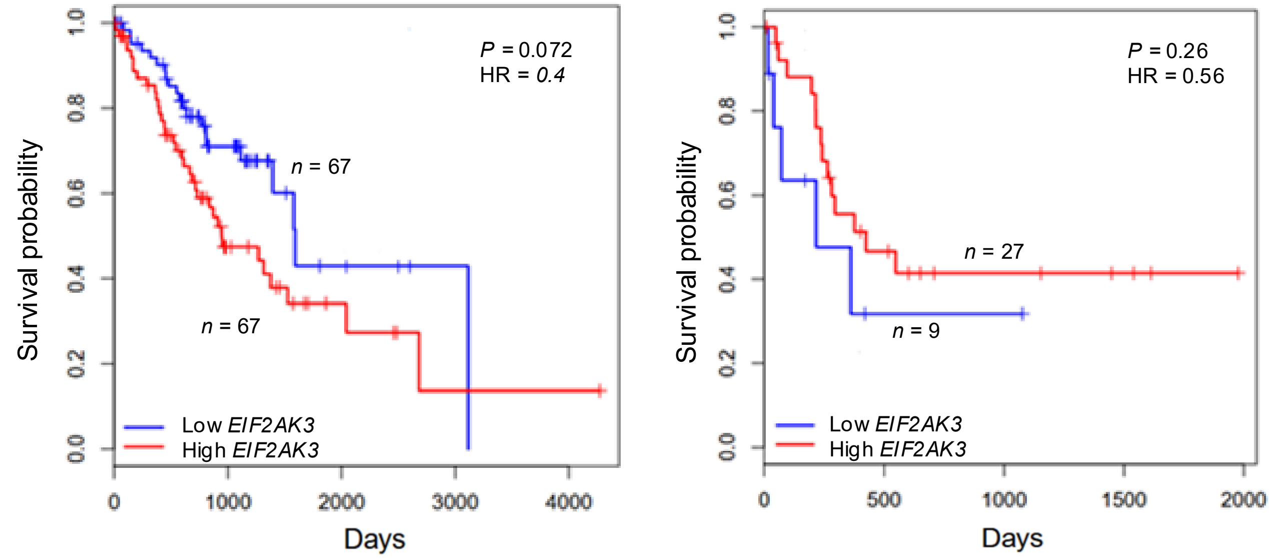

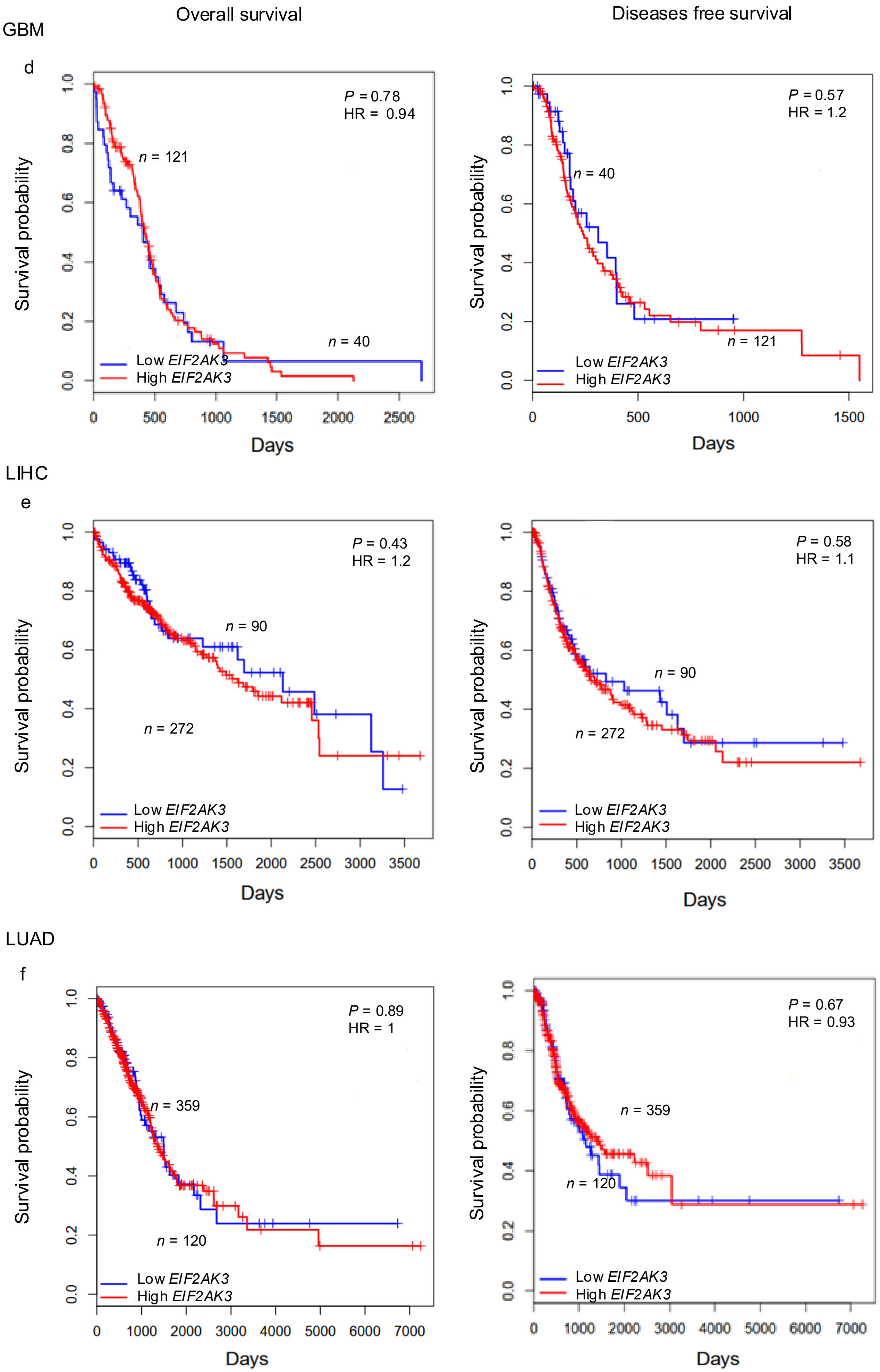

Overall survival
Diseases free survival
GBM
d
P = 0.78
HR = 0.94
n = 121
n = 40
Low EIF2AK3
High EIF2AK3
P = 0.57
HR = 1.2
n = 40
n = 121
Low EIF2AK3
High EIF2AK3
LIHC
e
P = 0.43
HR = 1.2
n = 90
n = 272
Low EIF2AK3
High EIF2AK3
P = 0.58
HR = 1.1
n = 90
n = 272
Low EIF2AK3
High EIF2AK3
LUAD
f
P = 0.89
HR = 1
n = 359
n = 120
Low EIF2AK3
High EIF2AK3
P = 0.67
HR = 0.93
n = 359
n = 120
Low EIF2AK3
High EIF2AK3
Survival probability
Days

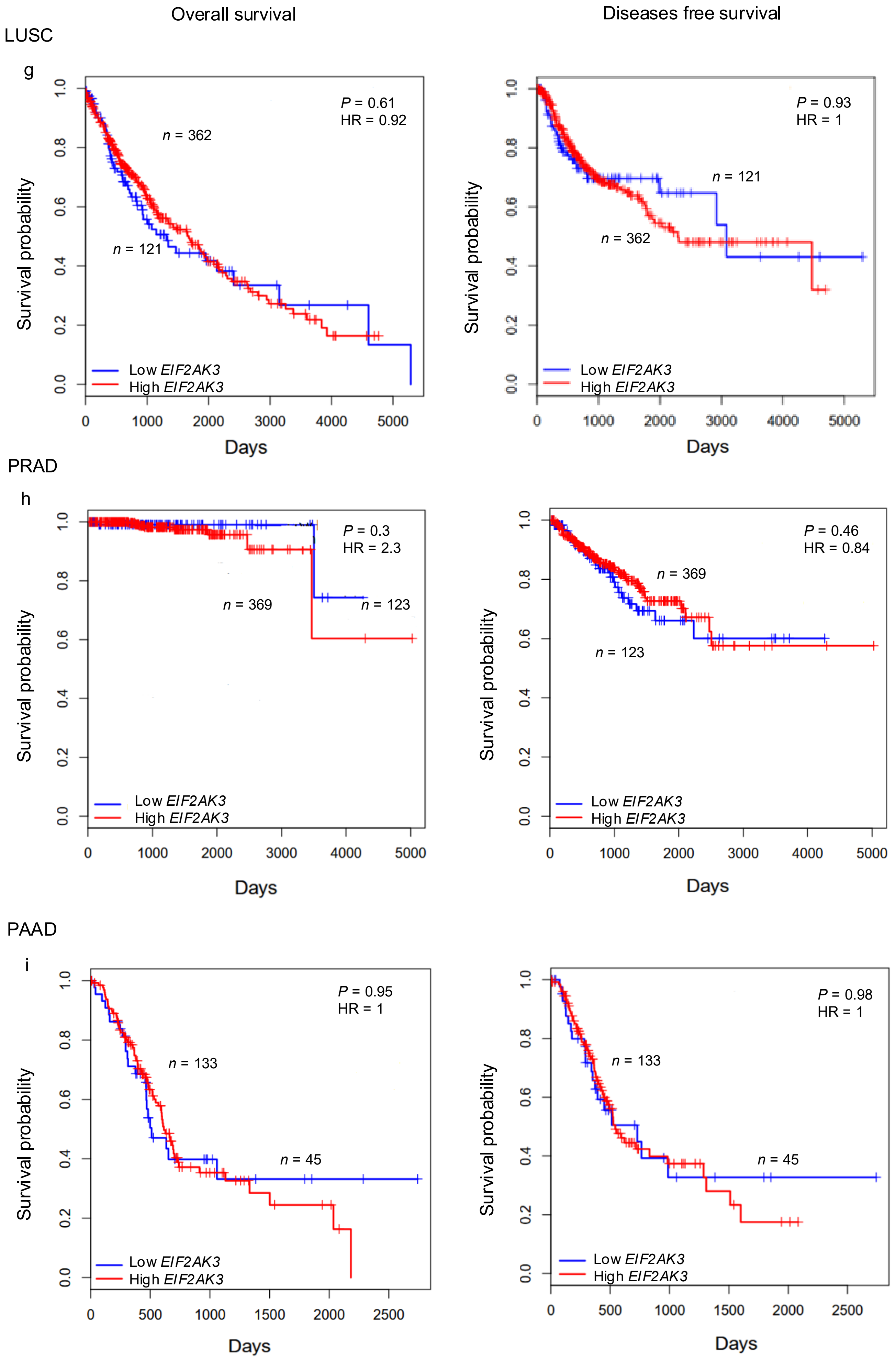

Overall survival
Diseases free survival
LUSC
g
P = 0.61
HR = 0.92
n = 362
n = 121
Low EIF2AK3
High EIF2AK3
Survival probability
Days
P = 0.93
HR = 1
n = 121
n = 362
Low EIF2AK3
High EIF2AK3
Survival probability
Days
PRAD
h
P = 0.3
HR = 2.3
n = 369
n = 123
Low EIF2AK3
High EIF2AK3
Survival probability
Days
P = 0.46
HR = 0.84
n = 369
n = 123
Low EIF2AK3
High EIF2AK3
Survival probability
Days
PAAD
i
P = 0.95
HR = 1
n = 133
n = 45
Low EIF2AK3
High EIF2AK3
Survival probability
Days
P = 0.98
HR = 1
n = 133
n = 45
Low EIF2AK3
High EIF2AK3
Survival probability
Days

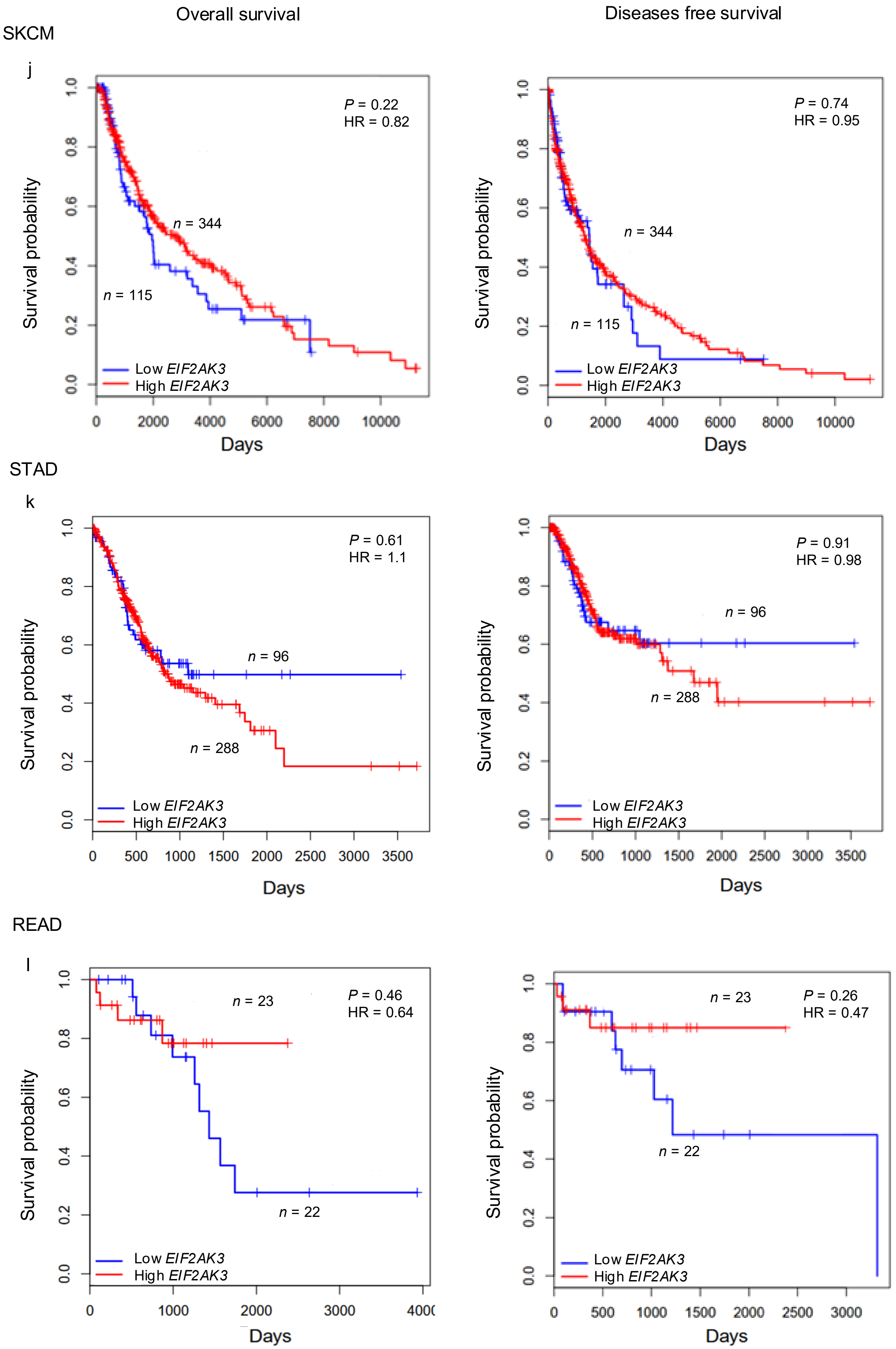

Overall survival
Diseases free survival
SKCM
j
P = 0.22
HR = 0.82
n = 344
n = 115
P = 0.74
HR = 0.95
n = 344
n = 115
STAD
k
P = 0.61
HR = 1.1
n = 96
n = 288
P = 0.91
HR = 0.98
n = 96
n = 288
READ
l
n = 23
P = 0.46
HR = 0.64
n = 22
n = 23
P = 0.26
HR = 0.47
n = 22
Survival probability
Days
Low EIF2AK3
High EIF2AK3

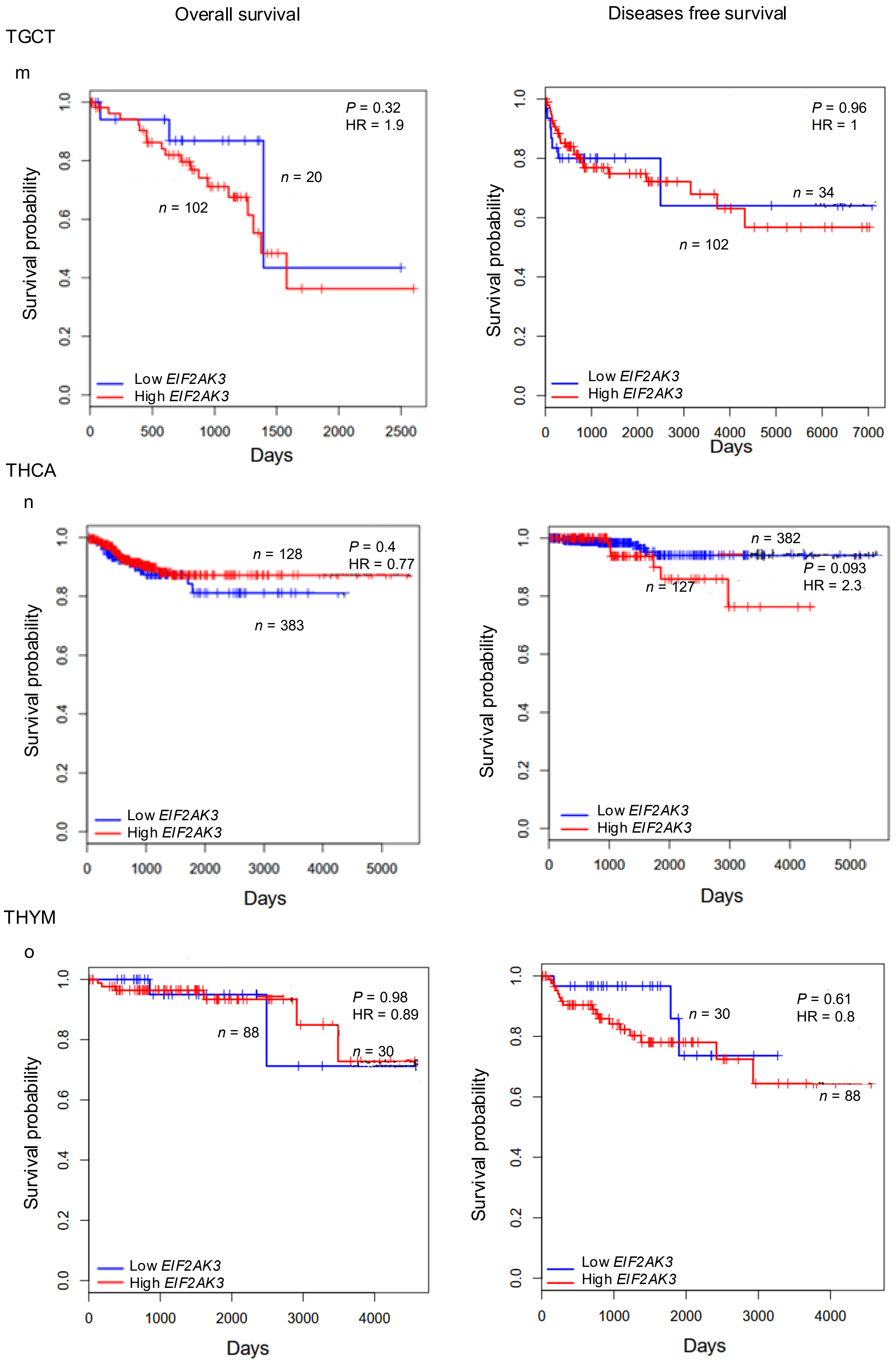
Overall survival
Diseases free survival
TGCT
m
P = 0.32
HR = 1.9
n = 20
n = 102
Low EIF2AK3
High EIF2AK3
P = 0.96
HR = 1
n = 34
n = 102
THCA
n
n = 128
P = 0.4
HR = 0.77
n = 383
n = 382
P = 0.093
HR = 2.3
n = 127
THYM
o
P = 0.98
HR = 0.89
n = 88
n = 30
P = 0.61
HR = 0.8
Survival probability
Days

Supplementary Fig. S9. Kaplan-Meier survival curve of overall survival (OS) and disease-free survival (DFS) for patients with (a) ACC, (b) COAD (c) CHOL, (d) GBM, (e) LIHC, (f) LUAD (g) LUSC, (h) PRAD, (i) PAAD, (j) SKCM, (k) STAD, (l) READ, (m) TGCT, (n) THCA and (o) THYM. The x-axis indicates survival time in days, and the y-axis shows the percentage of surviving patients. Numbers of patients in each group are displayed on the plots. *P*-values from the log-rank test and hazard ratios (HR) are indicated. Red lines represent the high *EIF2AK3* group, and blue lines represent the low *EIF2AK3* group.

a

CR Mutation Type	High Expression	Low Expression	Total
No mutation	4949	4949	9898
Missense	79	51	130
Multiple	2	6	8
Splice	4	1	5
Truncating	10	20	30

X^2	df	*P*-value
13.13	4	0.016

b

NCR Mutation Type	High Expression	Low Expression	Total
No variant	109	103	212
5'UTR	3	1	6
3'UTR	25	33	58
Intron	83	80	163

X^2	df	*P*-value
4.277	3	0.233

c

SCNA Mutation Type	High Expression	Low Expression	Total
Normal	4950	4950	9898
Amplification	3	0	3
Deep Deletion	0	3	3
Diploid	59	69	128
Gain	20	8	28
Shallow Deletion	3	6	9

X^2	df	*P*-value
12.92	5	0.057

Supplementary Table 1. Association between *EIF2AK3* expression level and (a) Coding region (CR) mutations, (b) NCR mutations and (c) Somatic copy number alterations (SCNA). Associations were evaluated using the chi-square test. Reported statistics include chi-square values (χ^2), degrees of freedom (df), and corresponding *P*-values.

a

Groups	Sum Squares	df	Mean Square	*F*-statistics	*P*-value
Age	9.62509	1	9.62509	8.571307	0.003452
Tumour Stage	9.668898	3	3.222966	2.870106	0.035194
Gender	14.54108	1	14.54108	12.94908	0.000328
Age*Tumour Stage	5.80121	3	1.933737	1.722026	0.160384
Age*Gender	0.552854	1	0.552854	0.492326	0.48297
Tumour Stage*Gender	9.527758	3	3.175919	2.828211	0.037242
Age*Gender* Tumour Stage	6.871671	3	2.290557	2.03978	0.106292

b

Groups	Sum Squares	df	Mean Square	*F*-statistics	*P*-value
Age	0.129839	1	0.129839	0.12806	0.72055
Tumour Stage	3.419288	3	1.139763	1.124146	0.338393
Gender	0.007627	1	0.007627	0.007522	0.930908
Age*Tumour Stage	5.287005	3	1.762335	1.738189	0.157704
Age*Gender	2.788609	1	2.788609	2.750402	0.097639
Tumour Stage*Gender	1.967516	3	0.655839	0.646853	0.585086
Age*Gender* Tumour Stage	1.062147	3	0.354049	0.349198	0.789746

Supplementary Table 2. Association of *EIF2AK3* gene expression with ages, genders and tumour stages. (a) Tumours with high *EIF2AK3* expression, (b) Tumours with low *EIF2AK3* expression. Analysis of covariance (ANCOVA) results are presented as sums of squares, degrees of freedom (df), mean squares, *F*-statistics, and *P*-values.
